# Single tryptophan Y160W mutant of homooligomeric *E. coli* purine nucleoside phosphorylase implies that dimers forming the hexamer are functionally not equivalent

**DOI:** 10.1038/s41598-021-90472-4

**Published:** 2021-05-27

**Authors:** Marta Narczyk, Łukasz Mioduszewski, Aleksandra Oksiejuk, Maria Winiewska-Szajewska, Beata Wielgus-Kutrowska, Adrian Gojdź, Joanna Cieśla, Agnieszka Bzowska

**Affiliations:** 1 Division of Biophysics, Institute of Experimental Physics, Faculty of Physics, University of Warsaw, Pasteura 5, 02-093 Warsaw, Poland; 2 Faculty of Chemistry, Warsaw University of Technology, Noakowskiego 3, 00-664 Warsaw, Poland; 3 Institute of Biochemistry and Biophysics Polish Academy of Sciences, Pawińskiego 5a , 02-106 Warsaw, Poland; 4 Present Address: Faculty of Mathematics and Natural Sciences, Cardinal Stefan Wyszyński University , Wóycickiego 1/3 , 01-938 Warsaw, Poland; 5 Present Address: Nencki Institute of Experimental Biology, Polish Academy of Sciences, Pasteura 3, 02-093 Warsaw, Poland

**Keywords:** Biochemistry, Biophysics, Molecular biology

## Abstract

*E. coli* purine nucleoside phosphorylase is a homohexamer, which structure, in the apo form, can be described as a trimer of dimers. Earlier studies suggested that ligand binding and kinetic properties are well described by two binding constants and two sets of kinetic constants. However, most of the crystal structures of this enzyme complexes with ligands do not hold the three-fold symmetry, but only two-fold symmetry, as one of the three dimers is different (both active sites in the open conformation) from the other two (one active site in the open and one in the closed conformation). Our recent detailed studies conducted over broad ligand concentration range suggest that protein–ligand complex formation in solution actually deviates from the two-binding-site model. To reveal the details of interactions present in the hexameric molecule we have engineered a single tryptophan Y160W mutant, responding with substantial intrinsic fluorescence change upon ligand binding. By observing various physical properties of the protein and its various complexes with substrate and substrate analogues we have shown that indeed three-binding-site model is necessary to properly describe binding of ligands by both the wild type enzyme and the Y160W mutant. Thus we have pointed out that a symmetrical dimer with both active sites in the open conformation is not forced to adopt this conformation by interactions in the crystal, but most probably the dimers forming the hexamer in solution are not equivalent as well. This, in turn, implies that an allosteric cooperation occurs not only within a dimer, but also among all three dimers forming a hexameric molecule.

## Introduction

Purine nucleoside phosphorylases (PNP, E.C. 2.4.2.1.) are ubiquitous enzymes of the purine salvage pathway enabling the cell to recover purine bases and ribose-1-phosphate from used purine nucleotides^1^. PNPs catalyse the following reaction: β-purine nucleoside + orthophosphate ↔ purine base + α-D-pentose-1-phosphate. Trimeric sub-family contains PNPs specific *vs.* 6-oxopurine nucleosides, and such enzymes are found in mammals and in some microorganisms, while hexameric PNPs present mainly in prokaryotes are less specific and accept many substituents at position 6 of the purine ring^1^. Both subfamilies are potential targets for antitumour, immunosuppressive, and/or antiparasitic agents^1–3^. Differences in specificity between trimeric mammalian PNPs and hexameric, less specific PNPs offer a basis for enzyme-activating prodrug gene therapy by empowering selective killing of tumour cells expressing the *E. coli* PNP gene^4–7^. These potential medical applications of PNPs encourage researchers to study their molecular mechanism and to search for their potent, selective and membrane permeable inhibitors.

**Figure 1:**
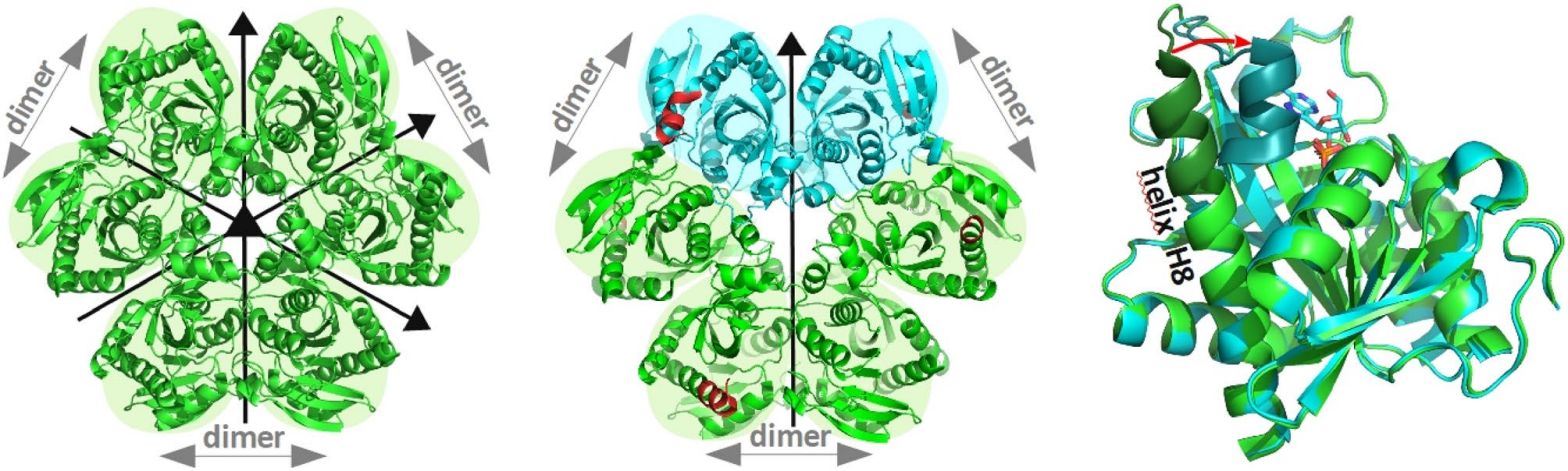
Left panel: Schematic view of the *E. coli* PNP in the apo form (PDB 1ECP^8^). All dimers are symmetrical, as all subunits have the same, elongated structure of the helix H8, resulting in the open conformation of the active site. The overall structure of the enzyme may be therefore regarded as a trimer of dimers as well as a dimer of trimers since the molecule has 32 point group symmetry. Middle panel: Schematic view of the *E. coli* PNP complexed with phosphate (PDB 4TS3^19^). In two subunits (here shown in cyan) binding of phosphate leads to the segmentation of the H8 helix and the partial closing of the active site pocket. Two dimers become unsymmetrical (shown here with cyan and green subunits), as they differ in the conformation of the N-terminal part of the H8 helix (shown in red). The third dimer (with green subunits) has both active sites in the open conformation, hence is symmetrical. The overall structure of the enzyme in this case is therefore a dimer of trimers. This kind of architecture is observed in the most of the crystal structures of *E. coli* PNP complexes available up to now, binary (with phosphate) and ternary (with phosphate and various nucleosides or purine bases). Right panel: Subunits in the open and closed conformation of the active site from the structure depicted in the middle panel, were overlaid to show differences in these two active site conformations: the segmentation of the helix H8 and the movement of its N-terminal part (shown in darker shades of green and cyan, respectively) and the loop towards the active site pocket (indicated by the red arrow).

Nevertheless, PNPs are also remarkable molecular machines and many molecular features that make them such efficient catalysts are still unclear. The biologically active form of the *E. coli* enzyme is a homohexamer, consisting of six monomers with an identical aminoacid sequence and, in the apo form, exhibiting also identical three-dimensional structure. However, the whole hexameric molecule has a three-fold symmetry, and can be described as a trimer of dimers. Moreover, each of the dimers has a two-fold symmetry, with the symmetry axis perpendicular to three-fold symmetry axis, so the apo enzyme may be regarded as a dimer of trimers as well (Fig. 1, left panel)^8^. Dimers, but not monomers, seem to have all the necessary features to conduct catalysis, because monomers within a dimer mutually donate two amino acids to complete the active site of the neighbour^9,10^. The earlier models describing enzyme kinetics and ligand binding have included two sets of kinetic and two binding constants, respectively, reflecting allosteric cooperation between subunits in a dimer^9,11,12^. Therefore, the hexameric (not simply dimeric) form adopted by this enzyme seems to be necessary solely in order to ensure stability of the enzyme molecule. This reason for homooligomerisation is observed for many other enzymes^14^ and we have already shown that it is important for this particular enzyme as well^15^.

However, most of the crystal structures of binary (with phosphate) and ternary (with phosphate and various nucleosides or purine bases) complexes of this enzyme available so far do not hold this three-fold symmetry^13,16–19^. Binding of a phosphate substrate, induces the conformational change leading to the closure of the active site pocket (Fig. 1, right panel). But this occurs only in some subunits. Two of the dimers are unsymmetrical, with one active site in the open and the other in the closed active site conformation (“open-closed” dimer), whereas the third dimer has both active sites in the open conformation (Fig. 1, middle panel). Since the closure of the active site is essential for catalysis^9,13^, only two “open-closed” dimers seem to be active.

**Figure 2:**
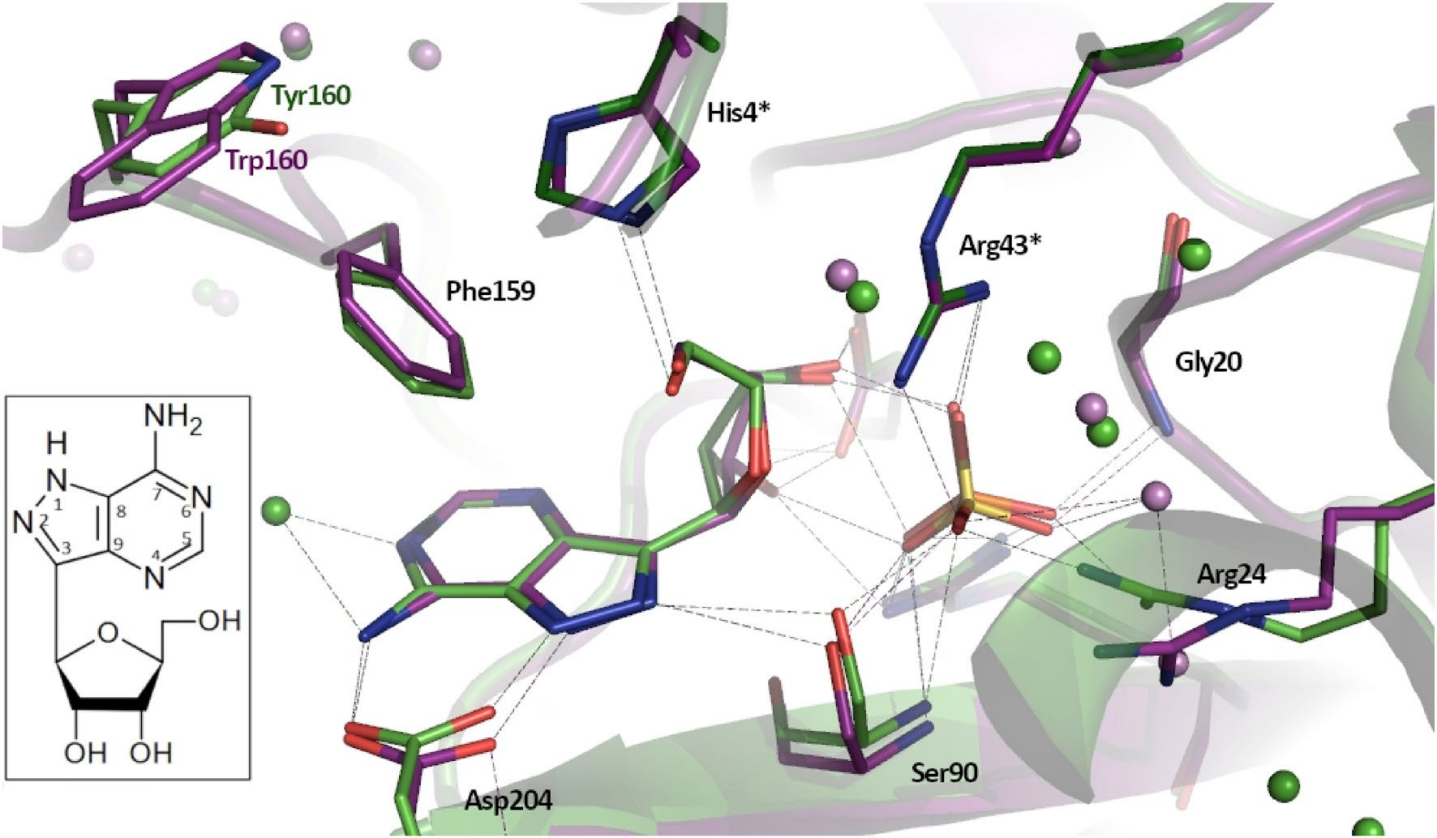
Comparison of active sites of the Y160W mutant (PDB 6XZ2, violet) and the WT PNP (PDB 4TS3^19^, green) complexed with formycin A and sulfate. Aminoacids forming the active site and the network of hydrogen bonds are shown. The residues His4 and Arg43 belonging to the neighbouring subunit in a dimer are marked with *. Inset: Formycin A, a structural analogue of adenosine, the natural *E. coli* PNP substrate. Due to the C–C glycosidic bond, that links the base and the pentose, formycin A is not a substrate for PNP, but an inhibitor, competitive *vs.* the nucleoside substrate. Note the difference in the base ring numbering when compared with the purine ring numbering.

Our earlier X-ray studies^13,19^ suggest that the open sites of the “open-open” dimer differ from the open sites of the “open-closed” dimer. In this study, we present the results of our research addressing the question whether the “open-open” dimer is forced to be symmetric by some interactions present only in the crystalline form, or it also differs from its neighbours in solution. In the latter case, its presence should be taken into account in the study of the molecular mechanism of catalysis. Therefore, we have examined in detail the properties in solution of the binary complex of *E. coli* PNP with phosphate, and of the ternary complex with phosphate and the nucleoside, formycin A, the structural analogue of the substrate, adenosine, and the potent inhibitor (formycin A, see Fig. 2, inset).

Analysis of the fluorescence titration data for complex formation of PNP with phosphate was the first indication that two types of dimers observed in the crystallographic structures were not just a crystallographic artefact. As the wild type PNP exhibits uniform changes of fluorescence upon phosphate binding, all titration curves, irrespectively of excitation and observation wavelength, have the same shape and are not visibly multiphase, two approaches were employed to further investigate the observed phenomena. First, a single-tryptophan Y160W mutant was engineered, with the mutation in a close proximity to the active site, but without significant interactions with ligands (Fig. 2). Since each subunit of the wild type PNP contains six tyrosine residues and no tryptophan^1^, this approach resulted in a protein with a significantly enhanced fluorescence response to ligand binding, and with retained catalytic and kinetic properties of the WT enzyme. Second, several experimental techniques were used to study the formation of binary and ternary complexes in order to explore the physical properties of the enzyme other than the intrinsic fluorescence of the protein.

## Results

### Y160W mutant shares typical features with the WT *E. coli* PNP

Analytical centrifugation confirmed that Y160W mutation did not affect the oligomeric state of the protein in solution. Virtually all protein molecules form a single band profile (Figure S1) with a sedimentation coefficient s_20,w_ = 7.80 which corresponds to the molecular weight of 150 kDa, in agreement with the hexameric assembly (6 × 25.970 = 155.8 kDa).

Crystallographic structure of the ternary complex of the Y160W mutant with two inhibitors, substrate analogues, sulfate ion and formycin A (PDB 6XZ2) shows that mutation has no significant influence on the secondary and ternary structure of the protein as well. Also, interactions of formycin and sulfate in the active site of the enzyme are comparable to those observed for the WT enzyme (Fig. 2). Complex crystallised in P 6_1_22 space group with the half of the hexamer in the asymmetric unit. All active sites are in the open conformation, consistent with the data on protein crystallisation in the absence of phosphate conditions^13^. Both ligands are in the same positions as in the active site of the WT PNP (PDB 4TS3, 4TS9^19^). Sulfate ion is bound in the hydrogen bond distance from Arg87, Arg43* (from the neighbouring subunit), Ser90, Gly20. The side of chain Arg24 points away from the active site, what is typical for the open conformation of the active site^9,13^. Ribose of formycin A is involved in hydrogen bonds with sulfate ion, Glu181 and His4* (from the neighbouring subunit), whereas aglycon—with Asp204 and Ser90.

The effect of the Y160W mutation on catalytic properties of the enzyme was checked by measuring the specific activity of the mutant *vs.* typical PNP substrates (Table 1), by collecting full kinetic data for guanosine, 7-methylguanosine and phosphate as variable substrates (Table 2), and by measuring the effect on the catalysis of the typical inhibitor, formycin A, competitive *vs.* nucleoside. Activity of the WT PNP and the mutant towards natural and modified substrates is very similar, but kinetic constants tend to be slightly higher for the mutant than those observed for the WT enzyme. However, all reaction models are retained: Michaelis–Menten for the nucleoside substrate, two allosterically interacting active sites for phosphate as a variable substrate, and competitive inhibition of formycin A *vs.* nucleoside substrate. All these results justify the use of the Y160W mutant to study formation of *E. coli* PNP complexes with ligands.

**Table 1:** Enzymatic specific activity of the Y160W PNP mutant *vs.* natural substrates (inosine, adenosine and guanosine) and *vs.* 7-methylguanosine compared with the similar data for the WT *E. coli* PNP.

Substrate Mutant	Ino		Ado		Guo		m^7^Guo	
	[U/mg]	[%]	[U/mg]	[%]	[U/mg]	[%]	[U/mg]	[%]
WT*	103.2 ± 4.0	100	58.5 ± 5.9	100	60.4 ± 6.7	100	21.4 ± 1.6	100
Y160W	91.1 ± 8.9	88	65.6 ± 14.2	112	36.2 ± 2.0	60	23.2 ± 1.2	108

Data were obtained at pH 7.0, in 50 mM Hepes buffer, 25 °C, with 50 mM phosphate using spectrophotometric methods described in “Methods” section.

*From^13^.

**Table 2:** The summary of kinetic properties of the WT PNP and the Y160W mutant *vs.* natural substrates and *vs.* 7-methylguanosine.

Kinetic parameter	WT	Y160W
Model	Allostery Eq. (2)	Allostery Eq. (2)
Phosphate as variable substrate, 0.4 mM m^7^Guo		
K_m_ [μM]	15 ± 4*	18.7 ± 2.7
V_max_ [U/mg]	10.8 ± 0.7*	11.7 ± 0.4
a	267 ± 232*	390 ± 122
b	0.76 ± 0.06*	0.94 ± 0.05

Kinetic parameter	WT	Y160W
Model	Michaelis–Menten Eq. (1)	Michaelis–Menten Eq. (1)
Guo as variable substrate, 50 mM phosphate		
K_m_ [μM]	28 ± 4	128 ± 2
V_max_ [U/mg]	51.1 ± 2.3	42.8 ± 0.3

Kinetic parameter	WT	Y160W
Model	Michaelis–Menten Eq. (1)	Michaelis–Menten Eq. (1)
m^7^Guo as variable substrate, 50 mM phosphate		
K_m_ [μM]	27.2*	71.3 ± 3
V_max_ [U/mg]	22.7 ± 0.3	21 ± 1

Kinetic parameter	WT	Y160W
Model	Competitive inhibition Eq. (3)	Competitive inhibition Eq. (3)
Formycin A (m^7^Guo as variable substrate, 50 mM phosphate)		
K_i_ [μM]	5.3 ± 0.4	42 ± 4

Data were obtained at pH 7.0, in 50 mM Hepes buffer, at 25 °C, with 50 mM phosphate using spectrophotometric methods described in “Methods” section. Parameters were obtained from the global fitting and are shown together with their standard deviations.

*From ^19^, 250 μM m^7^Guo.

### Effects of enzyme-phosphate interaction on enzyme fluorescence

**Figure 3:**
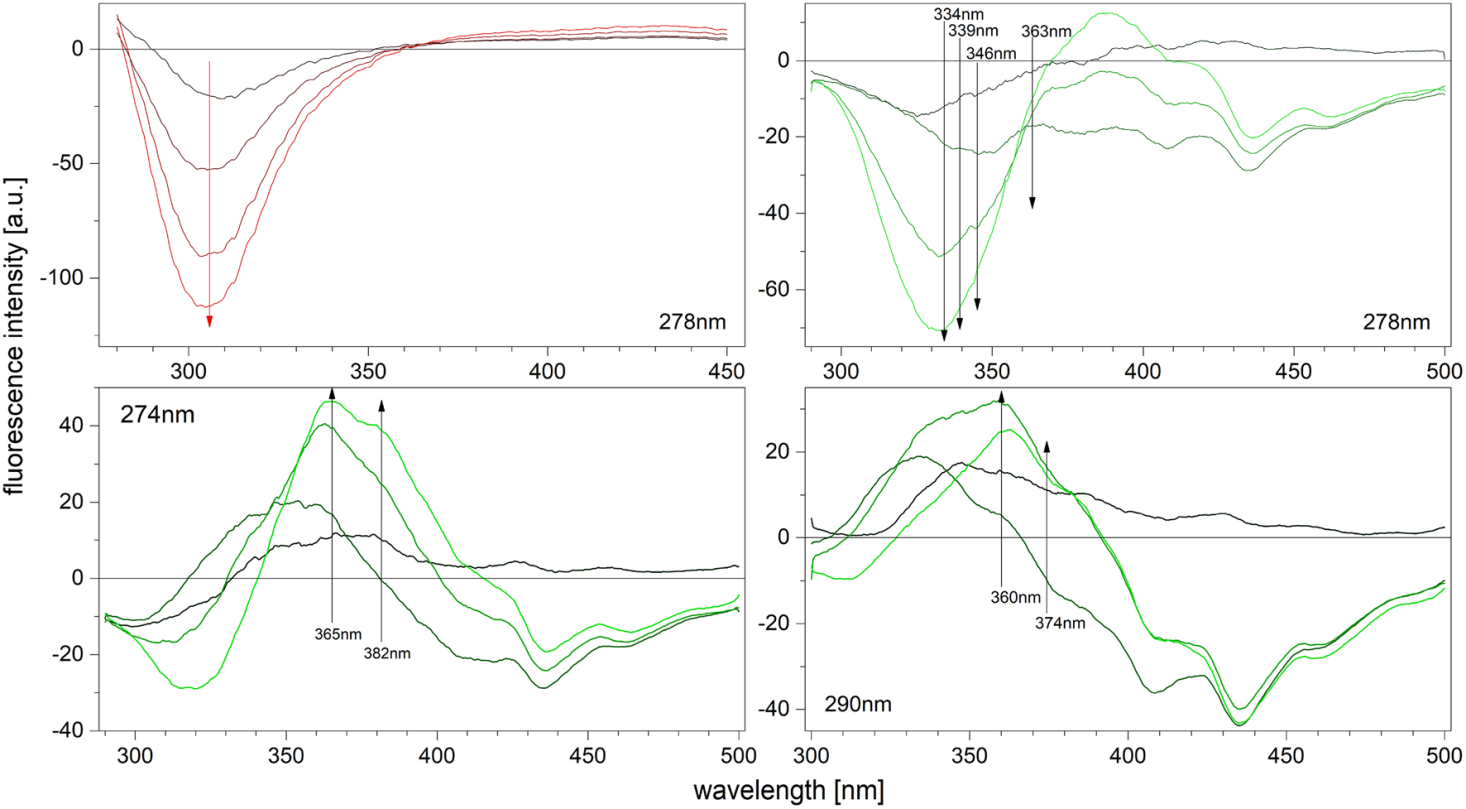
Differential fluorescence spectra of the *E. coli* PNP complexes with phosphate, left upper panel for the WT PNP (red), remaining graphs for the Y160W mutant (green), obtained at 25 °C, in 50 mM Tris/HCl buffer pH 7.6. The excitation wavelengths at which the spectra were measured are denoted on each graph. Phosphate concentrations were as follows: 10 μM, 100 μM, 1000 μM, 10 000 μM. The increase in phosphate concentration is marked with the gradient of the colour, black for the lowest, red (for the WT) or green (for the Y160W mutant) for the highest phosphate concentration. In the graphs for the Y160W mutant, the wavelengths chosen for the observation for each excitation wavelengths are marked by arrows. These data, together with the excitation wavelength shown on the graph, define conditions, in which titrations presented on Fig. 4, were performed. Direction of the arrows indicates only the direction of the phosphate concentration growth.

For the WT protein, as it has no tryptophan in the structure, increasing concentration of phosphate (Pi) in solution leads to only small quenching of the fluorescence without any significant changes in the shape of the protein fluorescence spectra, regardless of the excitation wavelength (Fig. 3). However, for the Y160W mutant changes in the protein fluorescence upon phosphate binding are much more diverse. Fluorescence spectra were collected with excitation at the maximum of the tyrosine (274 nm) and tryptophan (278 nm) absorption spectra, and at 290 nm where only tryptophan absorbs. Increasing the concentration of phosphate in the solution quenches or enhances the intrinsic fluorescence of the Y160W mutant (Fig. 3), and the effects strongly depend on the excitation wavelength and the region of the fluorescence spectrum. There are no isosbestic points, indicating the presence of more than two species (Fig. 3).

**Figure 4:**
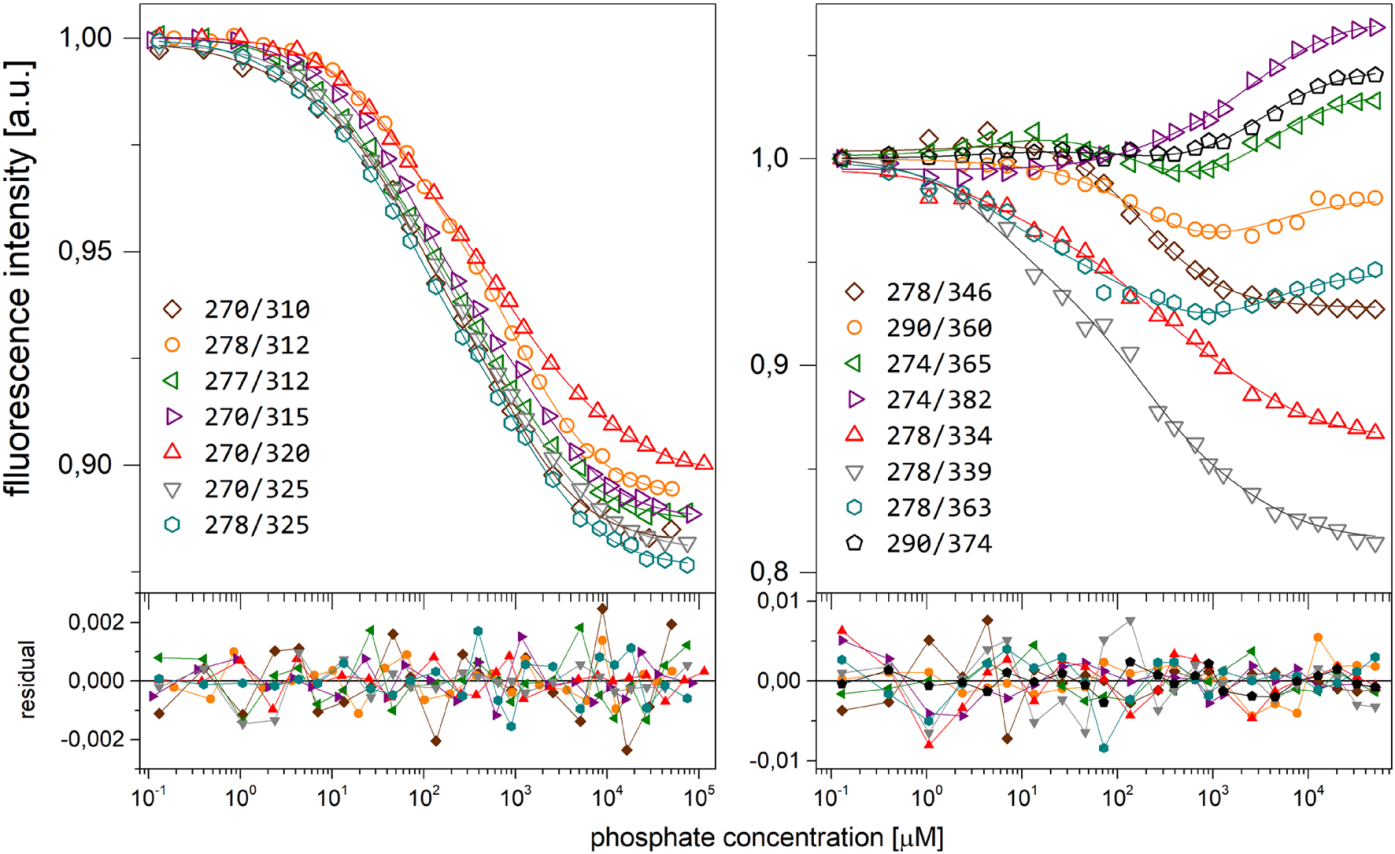
Fluorescence titration curves of the enzyme with phosphate: WT (left panel) and Y160W mutant (right panel). Data (points), global best fit model (lines) and residual plots are presented. Titrations were performed at 25 °C, in 50 mM Tris/HCl buffer pH 7.6 and in following excitation/observation wavelengths for the WT enzyme: 270/*3*10 nm (brown), 278/*3*12 nm (orange), 277/*3*12 nm (green), 270/*3*15 nm (violet), 270/*3*20 nm (red), 270/*3*25 nm (grey), 278/*3*25 (blue); and for the Y160W mutant: 278/346 nm (brown), 290/360 nm (orange), 274/365 nm (green), 274/382 nm (violet), 278/334 nm (red), 278/339 nm (grey), 278/363 nm (blue), 290/374 (black).

Based on the analysis of the differential spectra obtained for the Y160W mutant (Fig. 3) eight combinations of excitation and observation wavelengths were selected for titration measurements, in order to obtain curves with the greatest possible total change in fluorescence, and as many various titration curve shapes as possible. The typical series of titrations for both WT protein and Y160W mutant are shown in Figure 4. Even visual analysis of the curves obtained for the mutant suggests that binding is at least a three-step process, as some of the curves have more than one extremum (in the case of 290 nm/360 nm and 274 nm/365 nm excitation/observation wavelength, respectively). Curves were analysed globally as described in “Methods” section. Details of a discrimination analysis of the tested models are depicted in Table S1 in the Supplementary Information. For both WT protein and Y160W mutant three-binding-site model describes the data best, and values of dissociation constants obtained are shown in Table 3. As with kinetic parameters (Tables 1 and 2), constants for the Y160W mutant are slightly higher than for the WT protein, although within the 3σ test, K_d1_ and K_d3_ constants for both proteins are consistent, only the difference between constants K_d2_ exceeds the condition of compliance of this test. This may mean that introducing of tryptophan at position 160 has a not negligible effect on the ligand binding process described by the K_d2_ constant. Most importantly, however, the model describing the complex formation process for both proteins is the same, i.e. three binding sites with different affinity for the ligand are observed.

**Table 3:** Dissociation constants of the WT PNP and the Y160W mutant complexes with phosphate (Pi), and the WT PNP/Pi and the Y160W mutant/Pi complexes with formycin A (FA), determined by various experimental methods.

Enzyme	Titrant		Fluorescence titrations		Thermophoretic titrations		CD titrations	
			K_d_ ± SD	Confidence intervals	K_d_ ± SD	Confidence intervals	K_d_ ± SD	Confidence intervals
WT	Pi	K_d1_	(4.4 ± 1.1) μM	(1.8–6.6 ) μM	(7.0 ± 3.4) μM	(2.9–12.9) μM	(0.58 ± 0.80) μM	(< 0.07–2.12)* μM
		K_d2_	(84 ± 12) μM	(59–118) μM			(107 ± 30) μM	(76–144) μM
		K_d3_	(1.77 ± 0.14) mM	(1.47–2.23) mM	(1.64 ± 0.14) mM	(1.38–1.94) mM	(1.03 ± 0.29) mM	(0.76–1.46) mM
Y160W	Pi	K_d1_	(7.0 ± 1.4) μM	(4.8–10.9) μM	(3.20 ± 3.06) μM	(0.4–15.4) μM	(4.13 ± 3.77) μM	(0.3–9.7) μM
		K_d2_	(200 ± 30) μM	(179–409) μM			(128 ± 28) μM	(92–177) μM
		K_d3_	(3.2 ± 0.6) mM	(2.6–5.2) mM	(1.282 ± 0.079) mM	(1.12–1.45) mM	(2.33 ± 0.17) mM	(2.05–2.74) mM

Enzyme	Titrant		Fluorescence titrations		Thermophoretic titrations		Calorimetric titrations	
			K_d_ ± SD	Confidence interval	K_d_ ± SD	Confidence interval	K_d_ ± SD	Confidence interval
WT/Pi	FA	K_d1_	#	#	(0.0107 ± 0.0074) μM	(0.0005–0.0334) μM	(8.9 ± 1.9) μM	(5.5–10.8) μM
		K_d2_	#	#			(55 ± 19) μM	(40–81) μM
		K_d3_	#	#	(186 ± 38) μM	(117–303) μM	(812 ± 631) μM	(321–1615) μM
Y160W/ P_i_	FA	K_d1_	#	#	(0.137 ± 0.027) μM	(0.092–0.201) μM	(6.75 ± 0.30) μM	(4.01–10.10) μM
		K_d2_	#	#	(110 ± 23) μM	(29–246) μM	(148.9 ± 9.8) μM	(97.0–216.3) μM
		K_d3_	#	#	(1754 ± 774) μM	(994–5324) μM	(655 ± 37) μM	(412–1033) μM

Experiments were conducted at 25 °C, in 50 mM Tris/HCl buffer at pH 7.6, and in the case of titrations with formycin A in the presence of 50 mM phosphate buffer pH 7.6. Dissociation constants obtained from the global fitting (see “Methods” section) with standard deviations are shown, and also the confidence intervals at 3σ level (in parenthesis) .

*In this case the confidence interval is not closed from the bottom, as the lower bound is lower than the lowest ligand concentration used in titrations (0.007 μM).

^#^The strong absorption of FA makes fluorimetric titrations impossible even at moderate ligand concentrations.

### Binding of phosphate from MST and CD titrations

**Figure 5:**
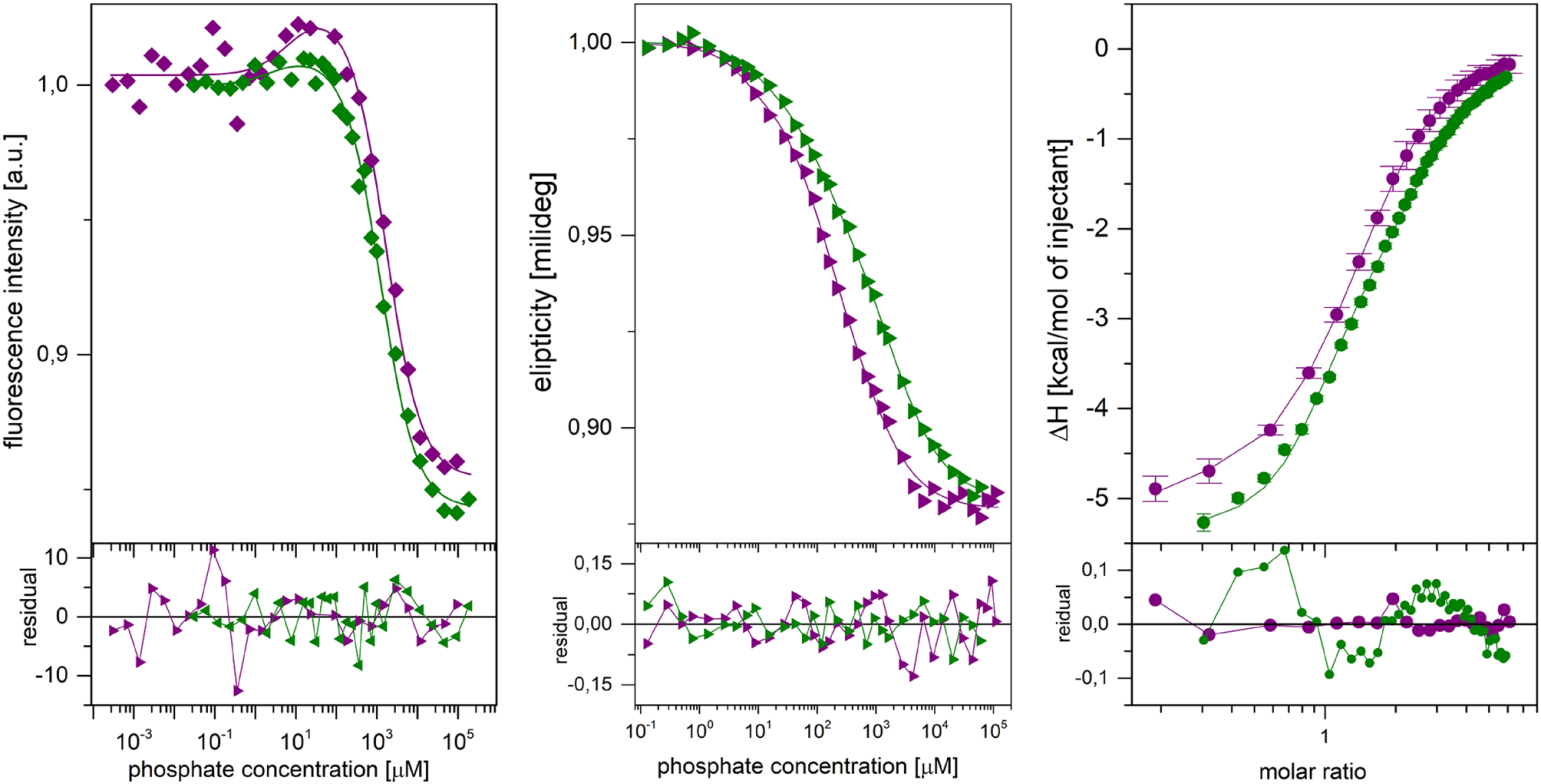
Thermophoretic titration curves of the enzyme with phosphate (left panel), CD titration curves of the enzyme with phosphate (middle panel), calorimetric titration curves of enzyme-phosphate complex with formycin A (right panel). On each panel data for the WT enzyme (violet) and the Y160W mutant (green) are presented: data (points), global best fit model (lines) and residual plots below. For the sake of clarity the number of data has been limited to one curve from each titration series. Complete data are shown in the Supplementary Information. Titrations were performed at 25 °C, in 50 mM Tris/HCl buffer pH 7.6. For each method and each protein variant the curve with the largest change of the measured physical property was chosen.

To confirm that there are three different types of binding sites, a series of thermophoretic (MST), calorimetric (ITC) and circular dichroism (CD) experiments were performed. The shapes of the binding curves obtained from MST titrations (Fig. 5 and Figure S2) are similar for WT PNP and Y160W mutant. Two-binding-site model fits the best to experimental binding curves (see details of the discrimination analysis, Table S1 in the Supplementary Information). The obtained values of the dissociation constants are shown in Table 3. Comparing these values with those obtained by fluorescence titrations, shows that dissociation constants determined by MST reproduce the strongest (K_d1_) and the weakest (K_d3_) constants obtained from fluorescence titrations analysis. K_d2_ constant is either undetectable by MST or is an artefact observed in fluorescence titrations.

To address this problem, calorimetric titrations (ITC) were employed, but due to the poor quality and low repeatability of results obtained, we decided to change the approach and perform CD titration instead. The near-UV CD spectra (230–300 nm) were collected to follow phosphate binding to the WT PNP and the Y160W mutant (Fig. 5 and Figure S4). The differential spectra of protein-phosphate complex *vs.* protein have the same shape for the WT protein and Y160W mutant, however introducing Trp at position 160 markedly changes the protein CD spectra, and the overall signal change is larger for the Y160W mutant (Figure S3). Titration curves were obtained by cross-secting the spectra in the wavelengths where the largest changes of the signal occur (see “Methods” section). The titration curves for both protein variants are best described by the three-binding-site model (see “Methods” section, Table S1 in the Supplementary Information for the details of a discrimination analysis). The values of dissociation constants derived from fitting this model are shown in Table 3.

### Interactions of *E. coli* PNP with phosphate

The results of titrations performed with the three techniques described above, i.e., fluorescence, thermophoresis and circular dichroism, show great similarity (Table 3). The values of the smallest dissociation constant (describing the strongest binding) determined by all methods are consistent with each other in the 3σ test. The same is true for the weakest constants. The third constant of 80–200 μM is unequivocally revealed by two methods, fluorescence and CD titrations. This suggests that in MST experiments this constant is simply undetectable. Possible reasons will be addressed in “Discussion” section. The results presented above indicate that the interaction of *E. coli* PNP with phosphate in solution requires three binding constants for the correct description, which is consistent with the crystallographic structure of the enzyme with phosphate (PDB 3OOH^13^). In this structure, the three-fold symmetry observed for the apo enzyme is broken, the active site pocket is closed in only two subunits, and as a result of this conformational change, the three dimers that build up the enzyme are no longer structurally equivalent.

### Binding of nucleoside analogue—ITC and MST titration studies

Binding studies of nucleoside by the WT PNP and the Y160W mutant were performed using the inhibitor, formycin A (FA, see Fig. 2, inset), a structural analogue of adenosine. Formycin A and formycin B, the analogues of the natural PNP substrates, adenosine and inosine, respectively, show strong absorption, and their fluorescence spectra overlap with that of the protein. Thus, fluorimetric studies employing natural emission of the protein and/or of the ligand, even at moderate ligand concentration, are impossible. Surprisingly, no changes in the CD signal were observed upon formycin A binding by either the WT or Y160W enzyme forms. Therefore, MST experiments and additionally isothermal calorimetric titrations were performed to characterize the binding of formycin A by PNP.

**Figure 6:**
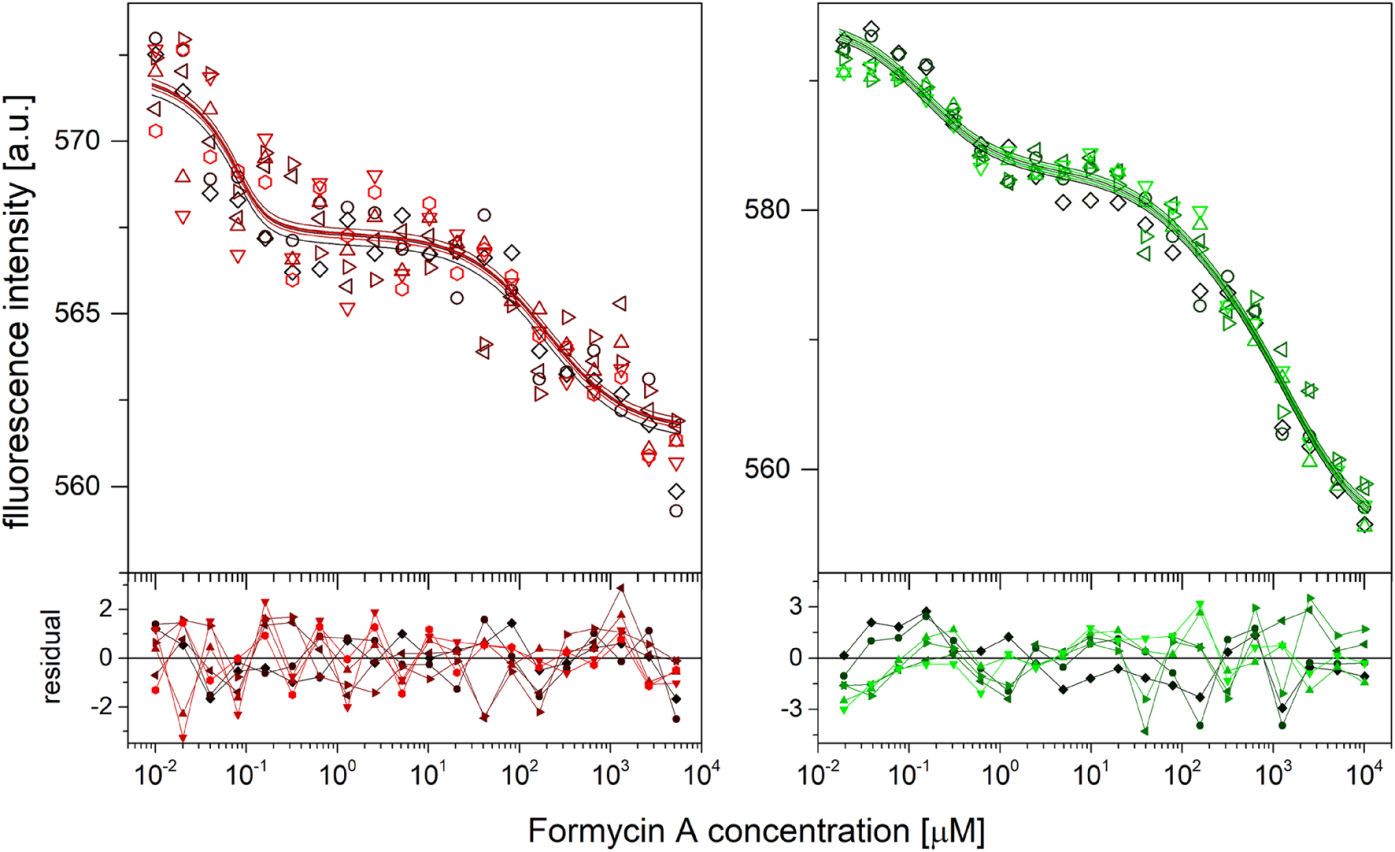
Thermophoretic titration curves of PNP-Pi complexes with formycin A: WT (left panel) and Y160W (right panel). Data (points), global best fit model (lines) and residual plots are presented. Titrations were performed at 25 °C, in 50 mM Tris/HCl buffer pH 7.6 and with the relative heating laser power at 80% for both WT protein and Y160W mutant. Seven repetitions of each titration were measured.

Calorimetric titrations were performed in series for the WT protein and for the Y160W mutant, and the enzyme concentration range used was 70–1300 μM (Fig. 5 and Figure S5). Global analysis of each titration series, for both proteins, were carried out in the Sedphat program^20,21^. Discrimination analysis (see “Methods” section and Table S1) in both cases pointed to the model with three binding sites as best describing titration data, and the values of dissociation constants derived from fitting of this equation are given in Table 3. In the case of the WT protein the errors of fitted parameters are relatively high, thus the values should be treated as estimates. In the case of thermophoretic measurements, even under the best conditions, the overall signal changes upon binding of formycin A molecules to the WT protein are relatively small, hence the analysis was not straightforward. The titration curves (Fig. 6) for both protein variants are clearly multiphase, indicating that the one-binding-site model is not consistent with the data. In the case of the WT protein, analysis of residual plots of the two-binding-site model suggests a more complex model (probability from Wald-Wolfowitz run test^22^ is 0.13, number of runs is below expected value). However, fitting the three-site-model returns the K_d1_ ~ 10^–8^ μM, while K_d2_ and K_d3_ are similar to those obtained from the two-binding-site model. The probability from the Wald-Wolfowitz run test is also very low (0.002) and the number of runs exceeds the expected value. The Akaike criterion^23^ indicates the two-binding-site model as the best (with Akaike weight equals 0.904). Thus, the two-binding-site model was chosen as best describing data for the WT protein. The values of dissociation constants are shown in Table 3. The analysis in the case of the Y160W mutant was unambiguous since the overall change in thermophoretic signal was significant (Fig. 6). All criteria point to the three-binding-site model as best describing the data obtained. The values of dissociation constants obtained by fitting this model are shown in Table 3.

Our recently published crystal structures of the WT PNP ternary complexes with phosphate and formycin A (PDB ID 4TS9, 4TS3^19^) show steps in nucleoside binding to the subunits of the enzyme hexamer. Crystallisation of the WT PNP complex with phosphate (at saturating concentration) and increasing concentration of formycin A (structure 4TS3) allowed to establish that in the first step, this inhibitor binds only in two closed active sites, which are located in adjacent subunits, but not belonging to the same dimer (as shown in Fig. 1, middle panel for the binary complex enzyme-phosphate). The position of formycin A in the active site of the Y160W mutant, in the structure presented in this study is virtually the same as in the active site of the WT protein (Fig. 2). In addition, the nucleoside is bound in only two active sites of the hexamer, in adjacent subunits but not belonging to the same dimer, exactly as in the partially occupied by formycin WT PNP structure 4TS3. Moreover, even in these active sites the ligand is not bound in all protein molecules, since the occupancy of the formycin A molecule was found to be 0.71. In the remaining active sites, faint electron density blobs are visible in the place usually occupied by a formycin molecule, suggesting that in a small percentage of the Y160W protein molecules the nucleoside may be bound in all active sites, as in the 4TS9 structure of the WT enzyme.

## Discussion

Early studies of hexameric *E. coli* PNP interactions with ligands, carried out over relatively narrow ligand concentration range, indicated that the kinetic and binding properties of this enzyme are well described by the set of two kinetic constants, and two binding constants, respectively^11,12^. The first crystal structure of the enzyme/ligand ternary complex^16^ revealed the presence of two conformations of the active sites, open and closed, in line with the results obtained for ligand binding and catalysis. However, in this structure only half of the hexamer was present in the asymmetric unit, hence the detailed analysis of the symmetry of the entire hexamer was not possible. The next published crystal structure^9^ of the *E. coli* PNP ternary complex, which has a full hexamer in the asymmetric unit, showed three similar dimers, each with one closed and one open active site. Thus, the architecture of the whole enzyme turned out to be a trimer of dimers, which seemed to be consistent with results obtained in solution^11,12^.

However, further crystallographic studies revealed that the latter structure^9^ was the only one with such symmetry, namely with all three dimers in the “open-closed” conformation. Subsequent X-ray structures of the WT *E. coli* PNP complexed with various ligands, when one of the ligands is phosphate^13,17–19^, do not show a trimer of dimers architecture, with the three-fold symmetry axis, but rather a dimer of trimers, having two-fold symmetry, as only two of the dimers are in “open-closed” conformation. In the third dimer both active sites remain open, breaking the three-fold symmetry. However, slight differences in the structure of open sites forming the “open-closed” dimers and open sites in the “open-open” dimer^13,19^ suggested, that there are three types of active sites, not just two. This discrepancy between the structural studies and the results obtained in solution was a rationale for undertaking experiments presented in this report.

Interaction of *E. coli* PNP with phosphate, investigated by fluorescence titrations over a broad range of ligand concentration yielded binding curves that are best described by a model with three dissociation constants, consistent with the symmetry of the enzyme molecule observed in the crystal. The engineering of a single tryptophan Y160W mutant, binding studies exploring also physical properties other than the intrinsic fluorescence of the protein and its complexes, as well as crystallisation of the mutant in a complex with substrates analogues were used to further investigate whether this type of symmetry actually exists when the protein is in solution.

Binding of phosphate was followed by fluorescence, circular dichroism and thermophoretic titrations. In the case of two former methods, for both WT PNP and Y160W mutant titration curves are best described by a model of three binding sites, while a thermophoretic titrations are best described by just two-binding-site model (Table 3). Comparing the values of the dissociation constants shows that the constants describing the strongest binding obtained by all three selected techniques, are consistent with each other (under the 3σ test). The same is true for the largest dissociation constant, describing the weakest binding. Two conformations of active sites, open and closed, with elongated and segmented helix H8, respectively, are visible in the crystallographic structures (Fig. 2), and at the same time opposite changes in the thermophoretic mobility are observed upon binding of phosphate to sites in the closed and in the open conformations, as depicted on Fig. 5, left panel and on Figure S2. Thus, the thermophoretic signal for the middle constant may simply be hidden between these two signals of opposite sign, which may account for the fact that the “middle” constant, K_d2_, is not detected by the MST. However, there is also another explanation. MST is a technique that relays on changes in protein structure, significant enough to influence the shape and hydration shell of the molecule, and thus the thermophoretic mobility. It is possible that the processes occurring in the protein molecule due to binding described by this constant just do not significantly affect these parameters. Fortunately, phosphate binding is associated with the significant arrest of the molecular motion, observed in the protein backbone fragments that are flexible in the ligand-free state, what was detected in the proton/deuter exchange experiments^24^. Such changes in the protein chain mobility are reflected in the intrinsic protein fluorescence and in the aromatic amino acids surrounding phosphate, giving rise to the CD signal for all three types of binding sites.

The next thing was to analyze binding of the nucleoside substrate. Calorimetric studies of binding by both WT protein and the Y160W mutant showed that three-binding-site model fits to the experimental titration curves the best. In the case of thermophoretic titrations, the two-binding-site model and the three-binding-site model better describe the data for the WT PNP and Y160W mutant, respectively. This difference for the two enzyme variants should rather be attributed to the better quality of titration curves obtained for the mutant, rather than to the actual difference in the binding model, since the total change in thermophoresis signal upon ligand binding is much larger and therefore the signal to noise ratio is better yielding the more “featured” curves (Fig. 6). Hence, three-binding-site model describing complex formation with the nucleoside seems valid for both protein variants.

The structure of the ternary complex of the Y160W mutant with sulfate and formycin A described in this study has a nucleoside bound only in two active sites per hexamer, located in the neighbouring subunits, but not belonging to the same dimer. Since phosphate was not present during the crystallisation, all active sites are in the open conformation. However, the distribution of occupied active sites makes this structure resemble the structure of the WT enzyme obtained at not saturating concentration of formycin A, captured as the first step in binding of this ligand by crystallographic “snapshots”. In the latter structure there is an analogous distribution of the occupied (and closed) active sites^19^. The distribution of the nucleoside molecules in the Y160W enzyme subunits indicates that although all active sites are in the same open conformation, they do not share the same affinity to the ligand and are therefore not equivalent. Three binding constants necessary to describe the thermophoretic titration curves and similarity of the curve describing the change in thermophoretic signal for both protein variants indicate that in the saturating phosphate concentration in crystallographic structures of the Y160W mutant, some active site pockets will most probably be closed, similarly as in the case of the WT enzyme.

**Figure 7:**
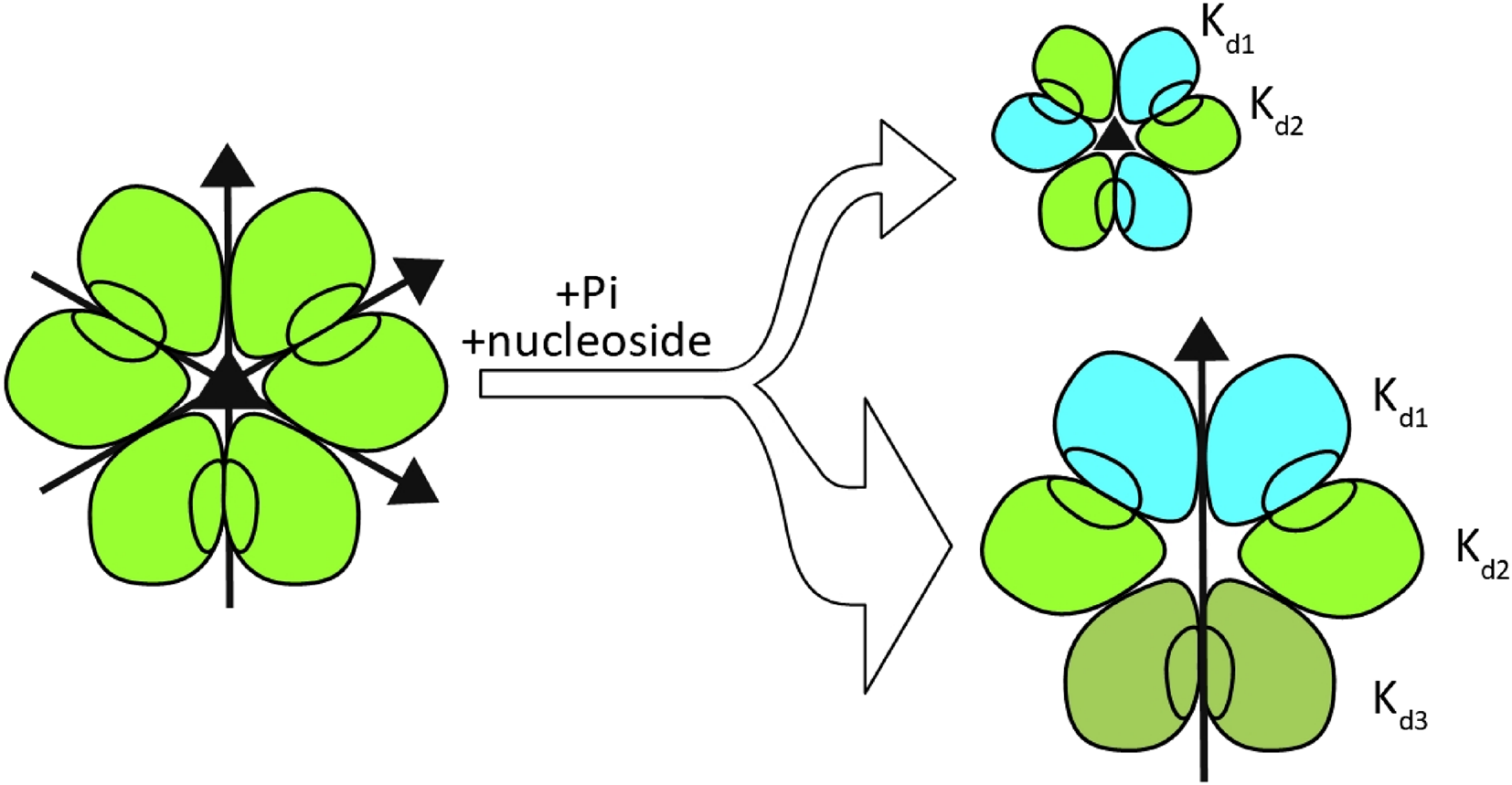
The schematic view of the *E. coli *PNP hexameric molecule, in the apo form, with the three-fold and the two-fold symmetry axes (left, based on PDB 1ECP^8^), and in the complexes with ligands, phosphate and nucleoside (right). In just one ternary complex of the *E. coli* PNP with its ligands (PDB 1K9S)^9^ the three-fold symmetry is retained (upper right), and all three dimers are with one monomer in the open (blue) and one in the closed (green) conformation of the active sites. The binary enzyme-phosphate complex, and the majority of various ternary enzyme-phosphate-nucleoside complexes show only two-fold symmetry (lower right, see also Fig. 1), as one of three dimers is symmetric (rotten green), hence different than two other unsymmetrical dimers (blue-green). The present study shows that this is more likely not a crystallographic artefact as also in solution the three-binding-site model is necessary to properly describe interaction of this enzyme with ligands, hence dimers are probably also functionally not equivalent.

The Y160W mutant turned out to be an excellent tool supporting the investigation of the interactions of hexameric *E. coli* PNP with ligands. The mutant not only yielded the expected more informative intrinsic protein fluorescence curves but also, unexpectedly, the calorimetric and thermophoretic titrations curves with a greater overall change in the measured parameter. The better signal-to-noise ratio and better reproducibility resulted in higher reliability of the obtained data. Using this tool, we have shown that breaking the three-fold symmetry, which is characteristic of most of the crystal structures of the hexameric *E. coli* PNP complexes with various ligands, is not a crystallographic artefact. Consistent with the two-fold rather than the three-fold symmetry of the overall enzyme architecture, this study indicates that the active sites in the open conformation of the “open-open” dimer most likely differ from the sites in the open conformation of the “open-closed” dimer, because three binding constants, not just two, are necessary to properly describe the interaction of *E. coli* PNP with both its substrates, the phosphate and the nucleoside (Fig. 7). Thus, also in solution, the three dimers making up the hexameric architecture of this enzyme are most likely not functionally equivalent. Two of them, with open and closed conformations of active sites, are catalytically active, while the third, “open-open” dimer is able to bind ligands but cannot undergo conformational change, consisting in closing of one active site, which is a necessary step in catalysis.

In this context it is worth mentioning once again that there is only one structure of the ternary complex of *E. coli* PNP with its ligands, in which the three-fold symmetry is maintained, and all three dimers occur with one monomer in the open and one in the closed conformation of the active sites^9^. The results described here, together with the presence of the aforementioned structure^9^, as well as our previous studies showing that dimers tend to form a hexamer because they are not stable as single molecules^15^, indicate that the molecular mechanism of *E. coli* PNP catalysis should take into account not only the cooperation between the two dimer-forming subunits, as indicated by the kinetic properties of this enzyme^11^ and initial ligand binding studies^9,12,13^, but also a cross-talk of all six hexamer subunits. The question thus arises whether under optimal conditions this cross-talk can lead to the “open-closed” conformation of all three dimers hence to a fully active enzyme, or the third dimer is doomed to be in the catalytically inactive “open-open” conformation. We tackle this problem in detail in our next study on this fascinating enzyme.

## Methods

### Chemicals

Formycin A was from Berry & Associates, Inc. (USA), adenosine, inosine, guanosine and xanthine oxidase from bovine milk (1 U/mg) were obtained from Sigma-Aldrich (USA). 7-methylguanosine (m^7^Guo) was synthesised from guanosine according to Jones’ and Robins' method involving methyl iodide^25^. This yields the preparation free from sulfate, which as an ion resembling phosphate could bias the results. All other chemicals were from Roth (Germany) or Sigma-Aldrich (USA), and were of the highest available purity. Background contaminant phosphate in buffers and other chemicals used in this study was measured spectrophotometrically using the method involving phosphomolybdate complex reduced by ascorbic acid^26^.

### PNP Y160W mutant

The *E. coli* strain with the pSE380 plasmid carrying *E. coli* PNP DeoD gene was a kind gift from Dr. Joanne L. Turnbull. The plasmid was a template in a site-directed mutagenesis performed with the use of Invitrogen GeneTailor Site-Directed Mutagenesis System (Invitrogen, USA) according to manufacturer protocol. Forward primer used for introducing the Y160W point mutation was as follows (the altered codon is underlined): 5′-TCTCCGCTGACCTGTTCTGGTCTCCGGACG-3′. The reverse primer was 5′-AGAACAGGTCAGCGGAGAACAGGTTACCCACC-3′. The synthesis of oligonucleotide primers and the sequencing of the mutated PNP gene were done by Genomed S.A. (Poland).

The *E. coli* DH5α-T1 cells containing the recombinant pSE380/PNPY160W plasmid were grown in Superbroth medium (2% peptone, 0.2% sodium phosphate (dibasic), 0.1% potassium phosphate (monobasic), 0.8% sodium chloride, 1.5% yeast extract, 0.2% glucose and 50 μg/ml ampicillin) at 37 °C with constant shaking (250 rpm) to OD_600_ = 0.42 and the culture was induced with 1 mM isopropyl β-D-1-thiogalactopyranoside (IPTG). The cells were grown for additional 5 h in the same conditions and harvested by centrifugation for 20 min at 4000×*g* at 4 °C.

The purification procedure was based on the previously published method^27^, but the additional chromatography step was added. The cell pellet obtained from 2.5 l of the bacterial culture was re-suspended in 50 ml of homogenizing buffer H (100 mM Tris–HCl, pH 7.6, 2 mM EDTA, 10% glycerol (v/v), 1 mM DTT, 50 mM NaCl, 0.4 mg/ml lysozyme and 0.015 mg/ml PMSF), incubated on ice for 30 min, sonicated and centrifuged for 20 min at 43,000×*g* at 4 °C. The supernatant was filtered through 4 layers of gauze, supplemented with an equal volume of 100 mM Tris–HCl buffer (pH 7.6) containing 2 mM EDTA, 10% glycerol (v/v) and 1 mM DTT (buffer A) and applied onto Q-Sepharose Fast Flow column (120 ml, GE Healthcare). The column was washed with 0.5 l of buffer A and subsequently with 360 ml of buffer A containing 0.1 M KCl. The enzyme was eluted with a 1.24 l linear gradient of 0.1–0.3 M KCl in buffer A. Fractions were assessed for PNP activity (using the spectrophotometric assay) and for the purity (using the SDS-PAGE). The purest fractions were pooled (210 ml), concentrated in Amicon stirred cell (Millipore) to 24 ml volume and supplemented with an equal volume of 2 M ammonium sulfate. The enzyme was applied onto Phenyl-Sepharose CL-4B (30 ml, GE Healthcare) equilibrated with 50 mM HEPES pH 7.6 containing 1 M ammonium sulfate. The column was washed with 200 ml of the same buffer and subsequently with the same buffer containing 0.8 M and 0.6 M ammonium sulfate (200 ml each). The enzyme was eluted with decreasing step gradient of ammonium sulfate in 50 mM HEPES pH 7.6, from 0.5 M to 0.1 M (100 ml each). The fractions containing PNP protein (named fraction 3 and fraction 4) were pooled separately, concentrated in the Amicon stirred cell, and dialysed against 10 mM HEPES pH 7.0 with two changes of dialysis buffer. The fraction 4, containing 60 mg of protein had slightly higher purity and therefore was used in all experiments described in this study. The protein was divided into small aliquots and stored frozen at − 80 °C until further use.

The recombinant WT enzyme was purified in the same way as described in our earlier publications^13,18,19^.

### Enzyme activity and kinetic studies

All activity measurements and kinetic studies were done in 50 mM Hepes/HCl buffer pH 7.0 at 25 °C as previously described for the WT *E. coli* PNP and its active-site mutants, using direct spectrophotometric assay for adenosine, guanosine and 7-methylguanosine as substrates, and a coupled assay with xanthine oxidase in the case of inosine^13,28,29^. Activity measurements were performed for each protein sample, before and after each experiment as a control of the state of the protein.

The classical Michaelis–Menten equation was fitted to the kinetic curves (i.e. initial rate of the reaction, v_0_, *vs.* initial substrate concentration, c_0_):

1 $${v}_{0}\left({c}_{0}\right)=\frac{{V}_{{\text{max}}1}{c}_{0}}{{c}_{0}{+ \text{K} }_{\text{M1}}}$$

If the analysis of residual plots suggested that a more complex model was necessary to describe the data properly, a model taking into account allosteric interaction between active sites was applied:

2 $${v}_{0}\left({c}_{0}\right)=\frac{\frac{2{V}_{\text{max1}}{c}_{0}}{{K}_{\text{M1}}}+\frac{2{\text{V}}_{\text{max2}}{c}_{0}^{2}}{{\text{K}}_{\text{M2}}^{2}}}{1+\frac{2{c}_{0}}{{K}_{\text{M1}}}+\frac{{c}_{0}^{2}}{{\text{aK}}_{\text{M1}}^{2}}}$$

where *K*_*M2*_ = *aK*_*M1*_ and *V*_*max2*_ = *bV*_*max1*_ characterizes a free active site when one binding site is already occupied by the ligands^30^. Coefficients *a* and *b* express the degree of cooperation between the subunits in a dimer, while constants *K*_*M1*_ and *V*_*max1*_ characterize the active sites of the enzyme when the neighbouring site is free.

A more complex model was used if there was a statistically significant decrease in the residual sum of squares as judged by the F test at 95% confidence level.

To determine inhibition model and inhibition constant for formycin A, rate of phosphorolysis with m^7^Guo as a variable substrate, at constant, 50 mM, phosphate concentration, was registered in the absence of the inhibitor and in the presence of two different concentrations of formycin. A global fitting to the whole data set was performed, using various models (competitive, uncompetitive, noncompetitive, mixed). As expected for the substrate analogue, competitive model described the data sufficiently well:

3 $${v}_{0}\left({c}_{0}\right)=\frac{{V}_{{\text{max}}1}{c}_{0}}{{c}_{0}{+ \text{K} }_{\text{M}}\left(1+{c}_{i}/{K}_{i}\right)}$$

where c_i_ is inhibitor concentration. Kinetic data were analysed using the GraphPad Prism program (Intuitive Software for Science, San Diego, CA, USA).

### Analytical ultracentrifugation

Sedimentation velocity experiments were conducted with an Optima XL-I (Beckman-Coulter Inc., Indianapolis, IN, USA) using the An-50Ti and An-60Ti analytical rotors, and double-sector 1.2-cm cells with Epon-charcoal centerpieces and sapphire windows. Partial specific volume of the Y160W mutant (from its amino acid composition), densities and viscosities of buffers were calculated using the Sednterp program^31^. Protein sample in 50 mM Tris/HCl buffer pH 7.6 and 0.2 M NaCl was equilibrated at 20 °C or at 4 °C, accelerated to 42,000 rpm (142,250 g) at the cell bottom and radial scans of the protein-absorption profile in the cell were measured at 5- or 7-min intervals. For the analysis, the continuous c(s) distribution model incorporated in the Sedfit program^32^ was used. The sedimentation coefficient s and the standard sedimentation coefficient s^0^_20,w_ were calculated, from which the molecular weight of the species present in the solution was derived, assuming that they have the same friction ratio (obtained as a parameter in the Sedfit program).

### Crystallisation, data collection and structure solution

The Y160W protein was crystallised in the 50 mM citrate buffer pH 5.2 and 14% ammonium sulfate (w/v) in the presence of 50 mM phosphate and/or 5 mM formycin A, PNP concentration was 90 mg/ml. Crystals were obtained at 18 °C by the hanging-drop vapour diffusion method. Prior to flash-freezing in liquid nitrogen crystals were soaked in a cryoprotectant solution containing 30% glycerol. Data were collected at the beamline P14 operated by EMBL Hamburg at the PETRA III storage ring (DESY, Hamburg, Germany), images were processed with the XDS^33^. The structure was solved by the molecular replacement with the Phaser, being a part of CCP4 suite^34^, and as a model PNP-WT (PDB 1ECP^8^) was used. The structure was refined with the Phenix.refine^35^ and Coot^36^. The X-ray data collection and refinement statistics are shown in Table 4. The structure was deposited in the Protein Data Bank (PDB 6XZ2).

**Table 4:** X-ray data collection and refinement statistics.

Data collection	
Space group	P 6_1_ 2 2
Cell dimensions	
a, b, c (Å)	120.47, 120.47, 239.80
α, β, γ (°)	90.00, 90.00, 120.00
Resolution (Å)	119.90–1.65 (1.68–1.65)
Total number of reflections	4,929,666 (189,722)
Total number unique	123,021 (5,869)
Rmerge	0.175 (1.483)
Rmeas	0.177 (1.507)
Mean I/σ(I)	14.7 (2.9)
Completeness (%)	99.8 (98.3)
Multiplicity	40.1 (32.3)

Refinement	
Resolution (Å)	95.83–1.65 (1.68–1.65)
Total number of reflections	122,926
Rfree reflections	6,119
Rwork /Rfree	0.174/0.189 (0.220/0.270)
Number of atoms	5,755
Macromolecules	5,412
Ligands	34
Water	309
Protein residues	711
RMSD bonds (Å)	0.018
RMSD angles (°)	1.435
Ramachandran favored (%)	97.02
Ramachandran allowed (%)	2.84
Ramachandran outliers (%)	0.14
Rotamer outliers (%)	0.35
Clashscore	1.93
Average B-factor	27.16

Statistics for the highest resolution shell are shown in parentheses.

### Fluorimetric titrations

All experiments were carried out on the Perkin-Elmer LS55 spectrofluorimeter. Fluorescence spectra were registered using in each case (protein or protein–ligand complex) several excitation wavelengths. Differential spectra were calculated by subtracting spectrum of a protein from the spectrum of the appropriate complex. The negative differential fluorescence intensity means that a decrease of fluorescence upon complex formation is observed. Titrations of the enzyme with phosphate were registered using several excitation and observation wavelengths combinations. When selecting specific wavelengths for such a combination, two criteria were used: (1) the overall change in the fluorescence signal and (2) the complexity of the shape of the titration as the analysis of the multi-phasic curves is more explicit than a uni-phasic curve analysis.

Path length for the excitation was 2 mm and for the emission was 5 mm (half of the cuvette dimensions). A continuous stirring was used during titrations. All measurements were performed at 25 °C. Enzyme concentration (as a monomer) was in the range of 0.5–2 μM. Molecular weight of the Y160W monomer used in all calculations was 25.970 kDa, and the molar extinction coefficient at absorption maximum ε_278nm_ = 8 940 M^−1^ cm^−1^, corresponding to ε^1%^_278 nm_ = 0.345^37^. Protein and ligand were dissolved in 50 mM Tris/HCl buffer pH 7.6, ligands stocks contained protein at the same concentration as in the reaction cuvette, as proposed by Breer et al.^38^, therefore, there was no protein dilution during titration and no correction is needed.

Fluorescence intensity of the WT protein is stable over time, but in the case of the Y160W mutant a slight quenching of fluorescence is observed during titration, which typically lasted about 30 min as the time between each point in the titration was 1 min. Furthermore, adding phosphate during titration stabilises the intrinsic fluorescence of the protein. Therefore, for each phosphate concentration, [P_i_], used in titrations (each titration point), fluorescence intensity *vs.* time was measured, and for each of them the linear fit was found to be sufficient to describe the data. Slopes of these lines, a([P_i_]), plotted *vs.* phosphate concentration, give a typical decay curve, showing signal stabilisation upon increasing concentration of phosphate. Thus corrected binding curves, F^cor^([P_i_]), are given by the following equation, in which F_n_([P_i_]) is the fluorescence intensity measured for the phosphate concentration [P_i_], and n is the number of a given titration point in the particular titration:

4 $${F}_{n}^{cor}\left(\left[{P}_{i}\right]\right)={F}_{n}\left(\left[{P}_{i}\right]\right)+{\sum }_{m=1}^{n}a\left({\left[{P}_{i}\right]}_{m}\right)$$

At pH 7.6 phosphate is mostly in a form of a double-negative ion (pKa 7.2)^39^, thus ionic strength, I, of the solution raises significantly during titration with this ligand. To check its influence on the intrinsic protein fluorescence, titrations with NaCl were performed, giving dependence F(I). For the WT protein F(I) was found to be constant up to the ionic strength of about 100 mM, and then a steep decrease is observed. Titration curves of the WT protein with phosphate were therefore measured up to 100 mM ionic strength of solution, which is enough to observe a plateau. For the Y160W mutant a more complex dependence F(I) was observed, influenced also by the titration conditions (excitation and observation wavelengths). The dependence of the fluorescence on the ionic strength observed for the WT protein suggests that there is no specific influence of the increasing ionic strength on the protein intrinsic fluorescence. Therefore, to correct for the increase of the ionic strength caused by phosphate, the fluorescence intensity measured during titration with this ligand, F^obs^([P_i_]) was divided by F(I), both obtained for the same ionic strength, to get fluorescence intensity curve, F^bind^([P_i_]) in which changes of the fluorescence observed are caused only by the phosphate biding to the enzyme. Both F^obs^([P_i_]) and F(I) were corrected for the time dependent fluorescence quenching, according to Eq. (4), prior to the F^bind^([P_i_]) calculation.

Titrations were performed in series (consisting of five to eight curves), and analysed globally using the DynaFit4 program^40^ (see below).

### Calorimetric titrations

The isothermal titration experiments were performed on the MicroCal iTC200 (Malvern) microcalorimeter. Before each experiment, protein solution was dialysed against 50 mM Tris/HCl buffer pH 7.6, and ligands were dissolved in a dialysate buffer, subsequently all samples were filtered using 0.22 μm pore diameter filters, and degassed. All experiments were carried out at 25°C. For the WT protein series of three calorimetric titrations, with protein concentration in the range of 70 to 800 μM, for the Y160W mutant series of four titration with protein concentration in the range of 300 μM to 1.3 mM were performed. Samples of each protein used for these series came from a single preparation. Ligand samples were 8–12 times more concentrated than the protein sample, in order to achieve a diverse ligand/protein molar ratio during titration and thus better explore binding curves^41^. In some ITC experiments, in order to sample the lower plateau region more accurately, several initial points were measured with less ligand volume added at each step than used in the subsequent titration points measurements. An example of the ITC data is shown in the Supplementary Information (Figure S6). Raw thermograms were analysed with NITPIC^43^. Global analysis of titration curves was performed with Sedphat^20,21^.

### MST experiments

Measurements were performed with Monolith NT115 (NanoTemper). Enzyme was labelled with NT647 fluorescent dye (NanoTemper) according to the protocol provided by the manufacturer. Labelled proteins show fluorescence excitation and emission maxima of approximately 650 and 670 nm, respectively. The series of titrations were performed at a constant protein concentration. Both proteins (WT and mutant), and ligands were dissolved in 50 mM Tris/HCl pH 7.6 buffer, measurements were performed at 25 °C. We have proved previously, that PNP-WT is stable over a broad range of temperatures^13^, thus temperature rise in the focal volume during MST experiment, typically 2–6 °C^42^ should have no significant influence on the protein activity and its biding capability. In the pre-experiments, it was carefully checked for both proteins, WT and Y160W mutant whether any sign of aggregation or inactivation occurs, and if mutant behaviour is similar to that of the WT enzyme. An example of the raw thermophoretic curves obtained with the relative heating laser power at 60% and 80% are shown in the Supplementary Information (Figure S7).

For experiments of the binary complex formation with phosphate, curves were measured at four different powers of the heating laser. Two to five titrations at each heating power were measured, and curves were averaged for a particular heating power. Global fitting was performed for a series of all four titration curves obtained at four heating laser powers. Protein concentrations in capillaries were 0.14 μM for the WT and 0.21 μM for the Y160W mutant. Ligand concentration was in the range of 3.5 nM—750 mM. The standard sixteen point curves were insufficient to test necessary ligand concentration range, thus measurements for two ligand concentration ranges were performed. Concentrations were selected so that three to four points from each range overlap, allowing to stitch obtained curves into one titration curve, covering entire required ligand concentration range. Measurements have been made for high concentrations of phosphate in order to investigate if for the concentration above about 50 mM, any additional binding constants can be observed. This is an area inaccessible in fluorescence titration due to the quenching of the protein fluorescence at such a high ionic strength of the solution. It was verified that so high ionic strength does not affect the fluorescence of the dye used to label the protein.

Experiments of the nucleoside analogue binding were performed in the presence of saturating concentration of phosphate, i.e. 50 mM. For both, WT PNP and Y160W mutant seven repetitions of each curve were measured, for a single power of the heating laser. The power of the heating laser at which the strongest change of the thermophoretic signal upon ligand binding occurs was selected, namely 80% for both enzyme variants. The raw MST traces, for two highest tested laser powers, 60% and 80%, are presented in the Supplementary information (Figure S7) Protein concentration in the capillary was constant and equal to 0.215 μM and 0.0751 μM for the WT protein and the Y160W mutant, respectively. Series of titrations were analysed globally using the DynaFit4 program^40^ (see below).

### CD titrations

Experiments were performed with Chirascan spectrophotometer (Applied Photophysics), in the near UV region (240–290 nm), thus in the region where aromatic amino acids absorb. Full spectra of the protein/complex were recorded, with 0.2 nm resolution and 2 s averaging per point. Enzyme concentration was in the range of 20–120 μM, the 50 mM Tris/HCl buffer pH 7.6 was used, ligands were dissolved in the same buffer. All measurements were performed at 25 °C. Upon addition of each volume of phosphate solution spectrum was recorded in the range 230–300 nm, in the amino acids absorption region, with 0.25 nm step and 2 s per point signal averaging. Spectra were smoothed with Savitzky-Golay algorithm^44^ using a fourth degree polynomial and eleven points in the averaging window. The wavelengths of the greatest signal change were determined by calculating differential spectra. These wavelengths were used to plot the titration curves. Global analysis of five to seven curves was performed with DynaFit4^40^.

### Global analysis of binding curves

Three models were taken into account in analysis of binding curves: model with one (first equation below), two (first two equations) and three binding sites.

$$\begin{aligned} &\text{P}+ \text{L} \Leftrightarrow \text{PL}\, \text{with}\,\text{K}_{\text{d1}}\\ &\text{PL}+\text{L} \Leftrightarrow \text{PLL}\,\text{with}\,\text{K}_{\text{d2}}\\ &\text{PLL}+\text{L} \Leftrightarrow \text{PLLL} \, \text{with}\,\text{K}_{\text{d3}} \end{aligned}$$

The model with one binding site is based on the assumption that all monomers are equivalent, and there is no cooperation between them. In the model with two binding sites the basic functional unit of the protein is a dimer, possible non-equivalence of binding sites or cooperativity between active sites in the dimer are taken into account (in the case of no cooperativity dissociation constants should be related statistically: K_d2_ = 4K_d1_), the dimers within hexamer are equivalent. The model with three binding sites is based on the crystallographic structures of binary and ternary complexes of PNP with ligands, where two of the dimers are in the “open-closed” conformation, but the third dimer is symmetrical, in the “open-open” conformation. Thus, active sites in the open conformation belonging to “open-closed” and “open-open” dimer may not be equivalent. This model takes into account possibility of cooperation between all monomers within a hexameric molecule of the protein (in the case of no cooperativity dissociation constants should be related statistically: K_d3_ = 9K_d1_, K_d2_ = 3K_d1_).

Titration series were analysed globally, always starting from the simplest, one-binding-site model. If the residual plots, judged visually and by Runs of Sign Test/Wald-Wolfowitz Runs Test (WWtest)^22^, suggested that one-binding-site model does not describe data well, the two-binding-site model was tested. Only if the analysis of the residual plots for the two-binding-site model, judged by the WWtest, suggested that still more complex model should be tested, the three-binding-site model was fitted, and the residual plots analysis was performed for the results. For all tested models the Akaike information criterion^23^ was also calculated. Generally both criteria used in the discrimination analysis were pointing the same model as best describing the data. The only exception was thermophoretic titration of the WT protein with formycin A, and it is described in details in the Results. Analysed models were fitted to the raw data, whereas graphs presented in this study were prepared from the normalised data and the fitted curves. The parameters of the fit are dissociation constants and responses of the system. Responses are defined as changes in a measured physical quantity related to the formation of subsequent protein–ligand complexes: intrinsic protein fluorescence intensity, fluorescence intensity of the dye, heat and ellipticity for fluorimetric, thermophoretic, calorimetric and CD titrations, respectively. Responses are normalised for the protein concentration present in the titration, thus giving molar coefficients.

Many titration curves obtained in this study are uniphasic. In the Supplementary Information (Figure S8) an example of such a uniphasic titration curve is shown (WT PNP titration with Pi, followed by the near-UV CD detection), with one-, two- and three-binding site models fitted. Respective residual plots are also included. Although the signal changes monotonically it is clear even on the basis of the visual inspection that the one-binding site model should be rejected. In turn, the residual plots indicate that the two-binding site model should be rejected as well.

### Accession number

Structural data are available in PDB database under accession number PDB 6XZ2.

## Supplementary Information

Supplementary Information.

## Abbreviations

PNP: Purine nucleoside phosphorylase
Y160W: PNP with Trp at position 160
WT: Wild type
Pi: Orthophosphate
Ino: Inosine
Guo: Guanosine
m^7^Guo: 7-Methylguanosine
Ado: Adenosine
FA: Formycin A
K_i_: Inhibition constant
K_m_: Michaelis constant
V_max_: Maximal velocity
