# Supplementary material for: Single tryptophan Y160W mutant of homooligomeric E. coli purine nucleoside phosphorylase implies that dimers forming the hexamer are functionally not equivalent
Source: Sci Rep. 2021 May 27;11:11144. doi: 10.1038/s41598-021-90472-4 (PMC8160210; doi:10.1038/s41598-021-90472-4)
Supplement: Supplementary file 1 — Supplementary Information. [file 41598_2021_90472_MOESM1_ESM.pdf]

Supplementary information

Single tryptophan Y160W mutant of homooligomeric *E. coli* purine nucleoside phosphorylase implies that dimers forming the hexamer are functionally not equivalent

Marta Narczyk[1], Łukasz Mioduszewski[1,@], Aleksandra Oksiejuk[1,#], Maria Winiewska-Szajewska[1,3], Beata Wielgus-Kutrowska[1], Adrian Gojdź[2], Joanna Cieśla[2], Agnieszka Bzowska[1]

[1]Division of Biophysics, Institute of Experimental Physics, Faculty of Physics, University of Warsaw, Pasteura 5, 02-093, Warsaw, Poland

[2]Warsaw University of Technology, Faculty of Chemistry, Noakowskiego 3, 00-664 Warsaw, Poland

[3]Institute of Biochemistry and Biophysics Polish Academy of Sciences, Pawinskiego 5a, 02-106 Warszawa, Poland

Present address:

[@]Faculty of Mathematics and Natural Sciences, Cardinal Stefan Wyszynski University, Woycickiego 1/3, 01-938 Warsaw, Poland

[#]Nencki Institute of Experimental Biology, Polish Academy of Sciences, Pasteura 3, 02-093 Warsaw, Poland

Figure S1.

Sedimentation coefficient distribution obtained for the Y160W *E. coli* purine nucleoside phosphorylase (PNP) mutant in the sedimentation velocity experiment with the absorption detection, at 42 000 rpm, at 20°C in 50 mM Tris buffer pH 7.6 and 0.2 M NaCl. Inset shows experimental data and fitted c(S) model, with residuals distribution on the lower panel.

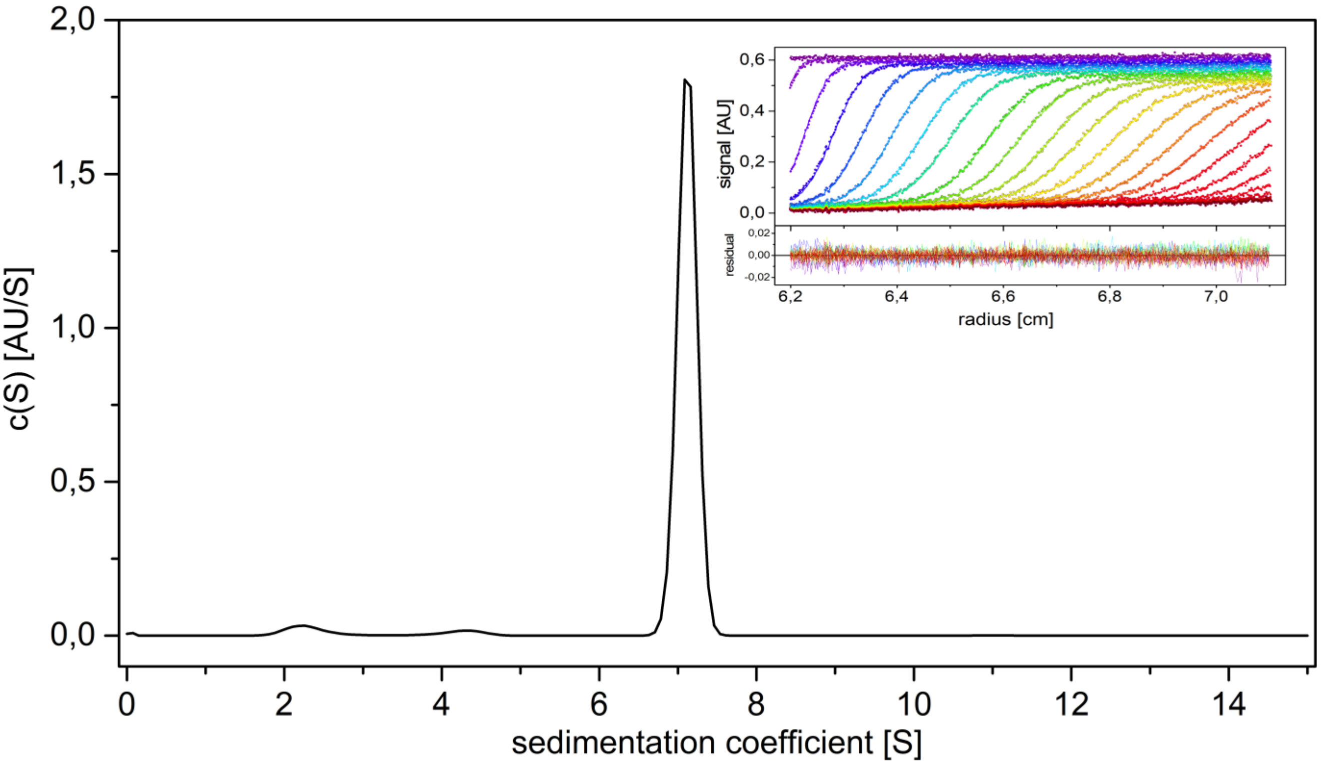

Figure S2.

Thermophoretic titration curves of the *E. coli* PNP with phosphate: WT (left panel) and Y160W mutant (right panel). Data (points), global best fit model (lines) and residual plots are presented. Titrations were performed at 25°C, in 50 mM Tris/HCl buffer pH 7.6 and with the following relative heating laser power for WT: 20% (brown), 40% (orange), 60% (green), 80% (violet), for Y160W 20% (brown), 40% (orange), 60% (green).

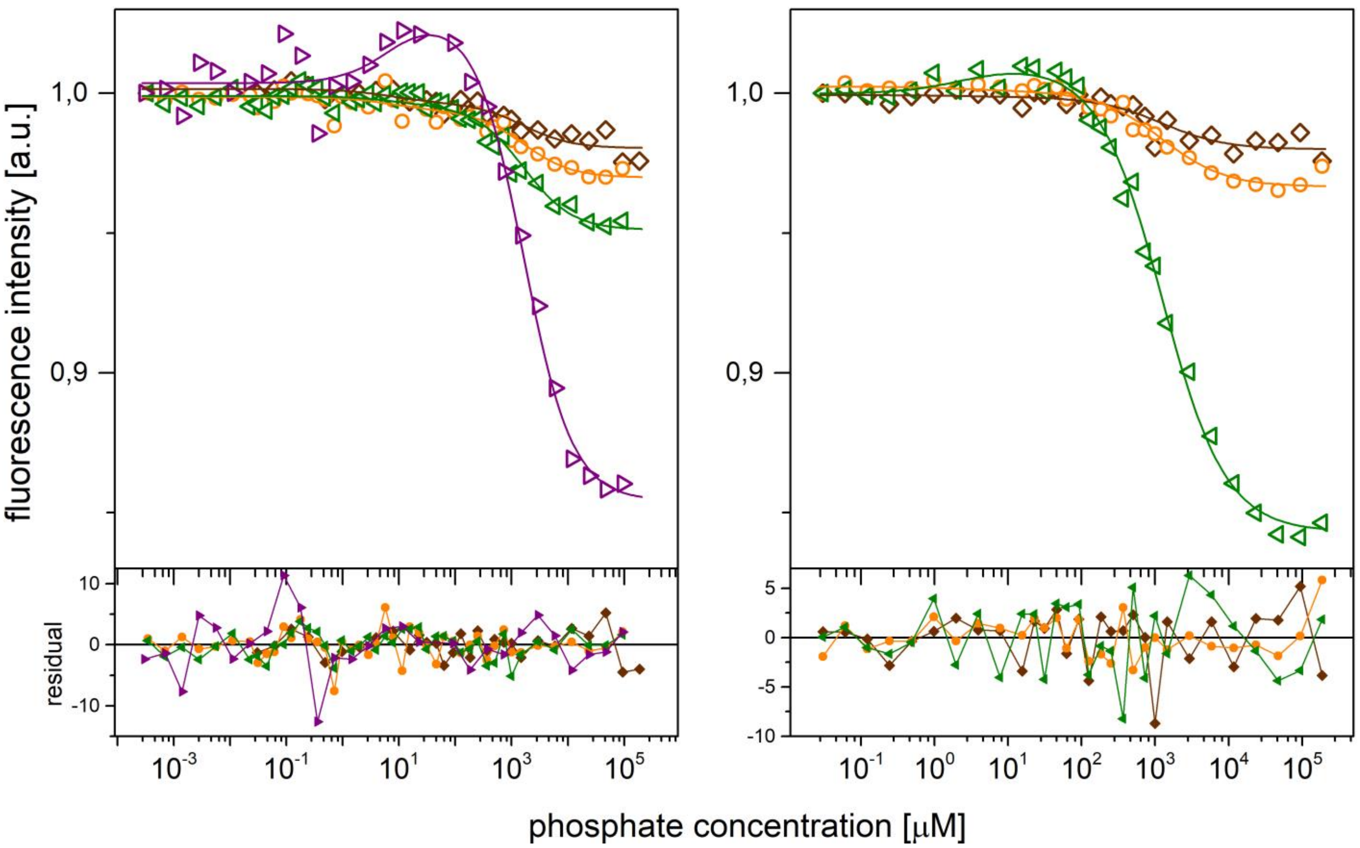

Figure S3.

Differential CD spectra of *E. coli* PNP complexes with phosphate, WT (left panel) and Y160W mutant (right panel), obtained at 25°C, in 50 mM Tris/HCl buffer pH 7.6. Phosphate concentration used was as follows: 12.63 µM, 137.1 µM, 1366 µM and 31 137 µM. Increase in phosphate concentration is marked with the gradient of the colour, black for the lowest, red (for the WT PNP) or green (for the Y160W mutant) for the highest phosphate concentration. In the insets CD spectra of the WT PNP (red) and Y160W mutant (green) are shown.

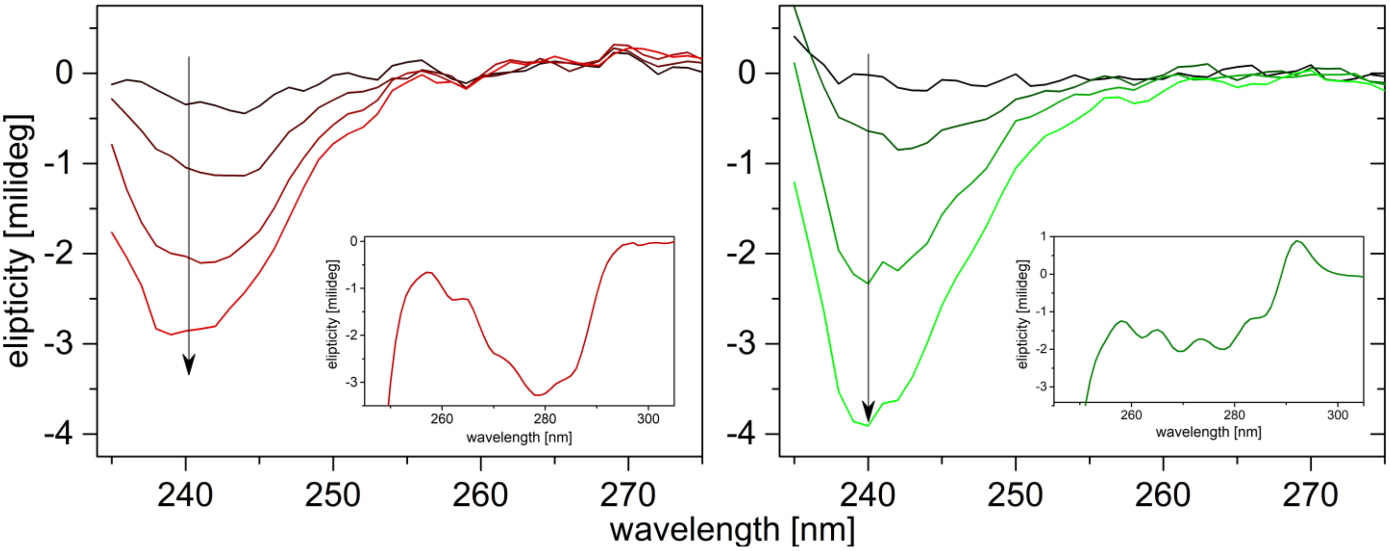

Figure S4.

CD titration curves of the WT *E. coli* PNP (left panel) and the Y160W mutant (right panel). Data (points), global best fit model (lines) and residual plots are presented. Titrations were performed at 25°C, in 50 mM Tris/HCl buffer pH 7.6 and in following observation wave-lengths for WT: 240nm (brown), 241.5 nm (orange), 242 nm (green), 242.5 nm (violet), 243 nm (red); for YW: 239.5nm (brown), 240 nm (orange), 240.5 nm (green), 241 nm (violet), 241.5 nm (red), 242 nm (gray), 242.5 (blue)

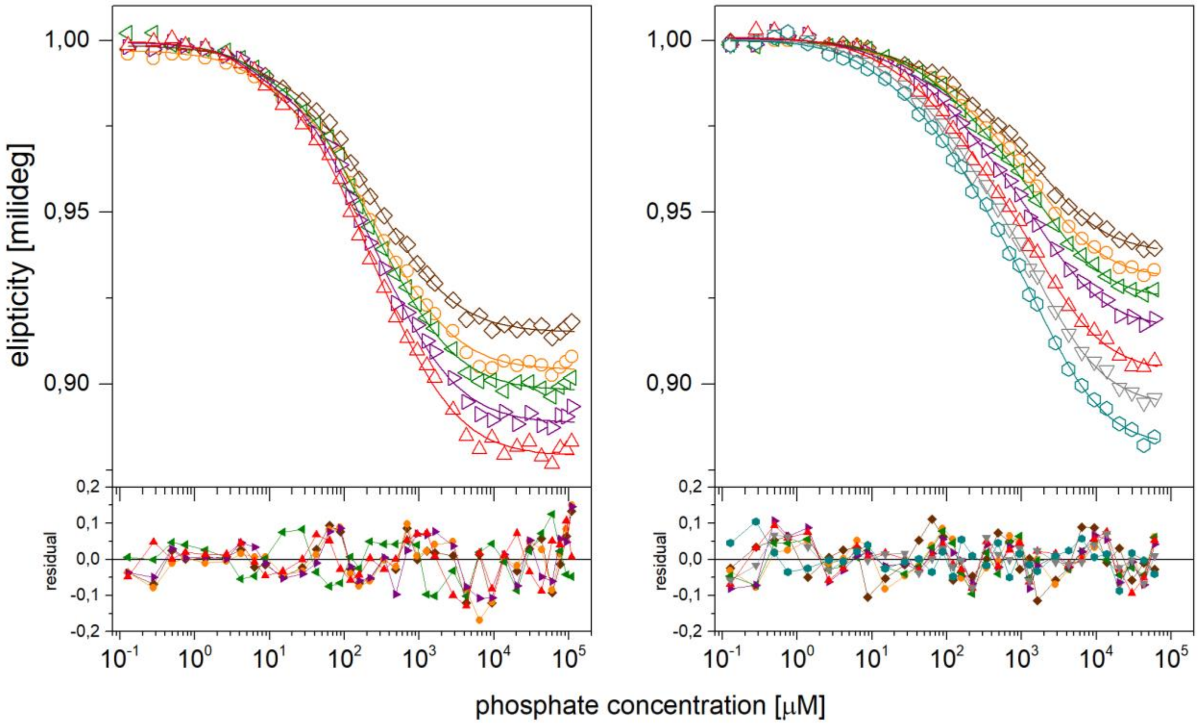

Figure S5.

Calorimetric titration curves of PNP-phosphate complexes with formycin A: WT (left panel) and Y160W mutant (right panel). Data (points), global best fit model (lines) and residual plots are presented. Titrations were performed at 25°C, in 50 mM Tris/HCl buffer pH 7.6 and with the following initial enzyme concentration/final ligand to protein molar ratio: for WT: 70.4 µM/14.8 (brown), 207.4 µM/6.1 (orange), 800.6 µm/2.5 (green) for YW: 301 µM/5.4 (brown), 486 µM/6.0 (orange), 933 µM/6.5 (green), 1.3 mM/7.0 (violet).

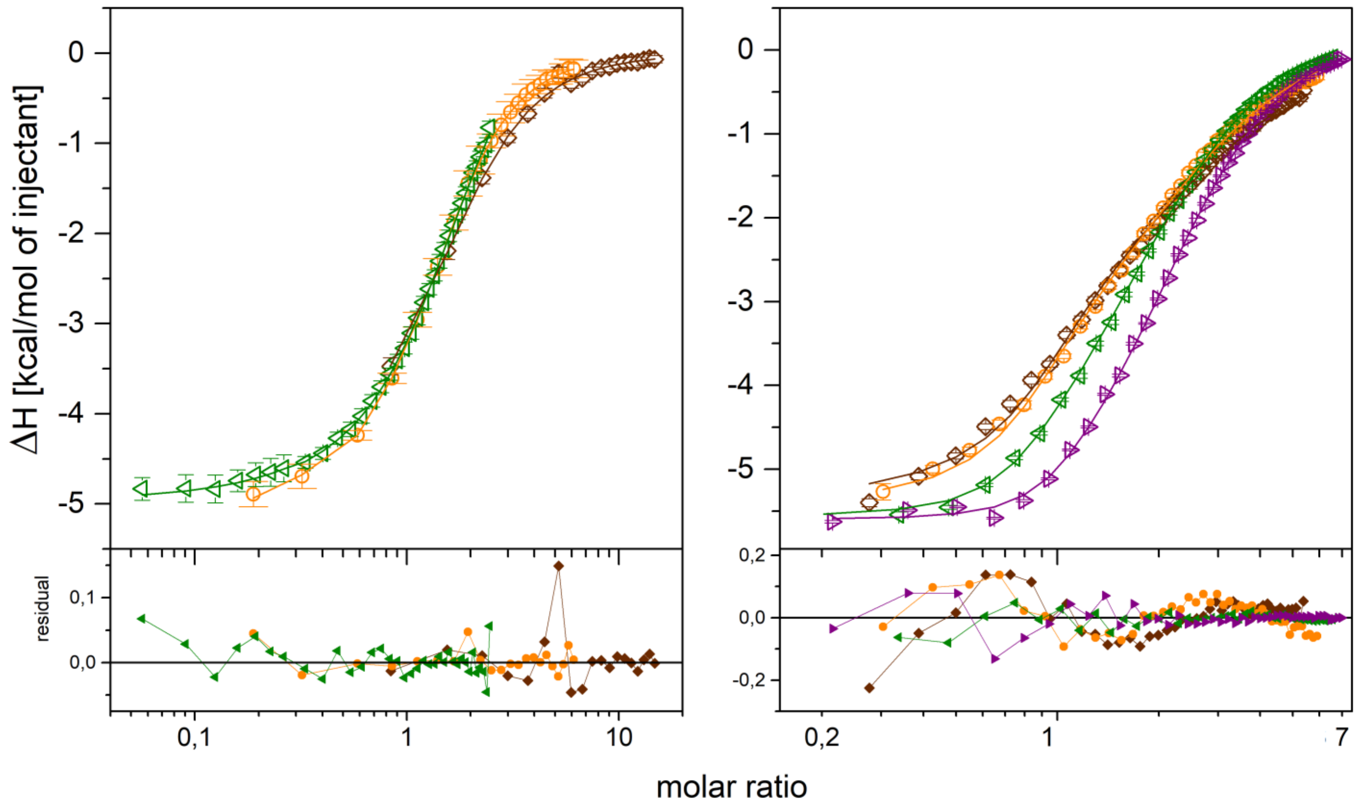

Figure S6

Thermograms of calorimetric titration of PNP-phosphate complexes with formycin A, normalised using NITPIC [43]. Titrations were performed at 25°C, in 50 mM Tris/HCl buffer pH 7.6, with initial enzyme concentration 207.4 µM for the WT PNP (upper panel) and 486 µM for Y160W mutant (lower panel). The drastic jump observed for the WT PNP titration is due to the change in the titrant volume added. This approach has been used in some ITC experiments to more accurately sample the lower plateau area, several initial points were measured with less ligand volume added in each step than further titration points.

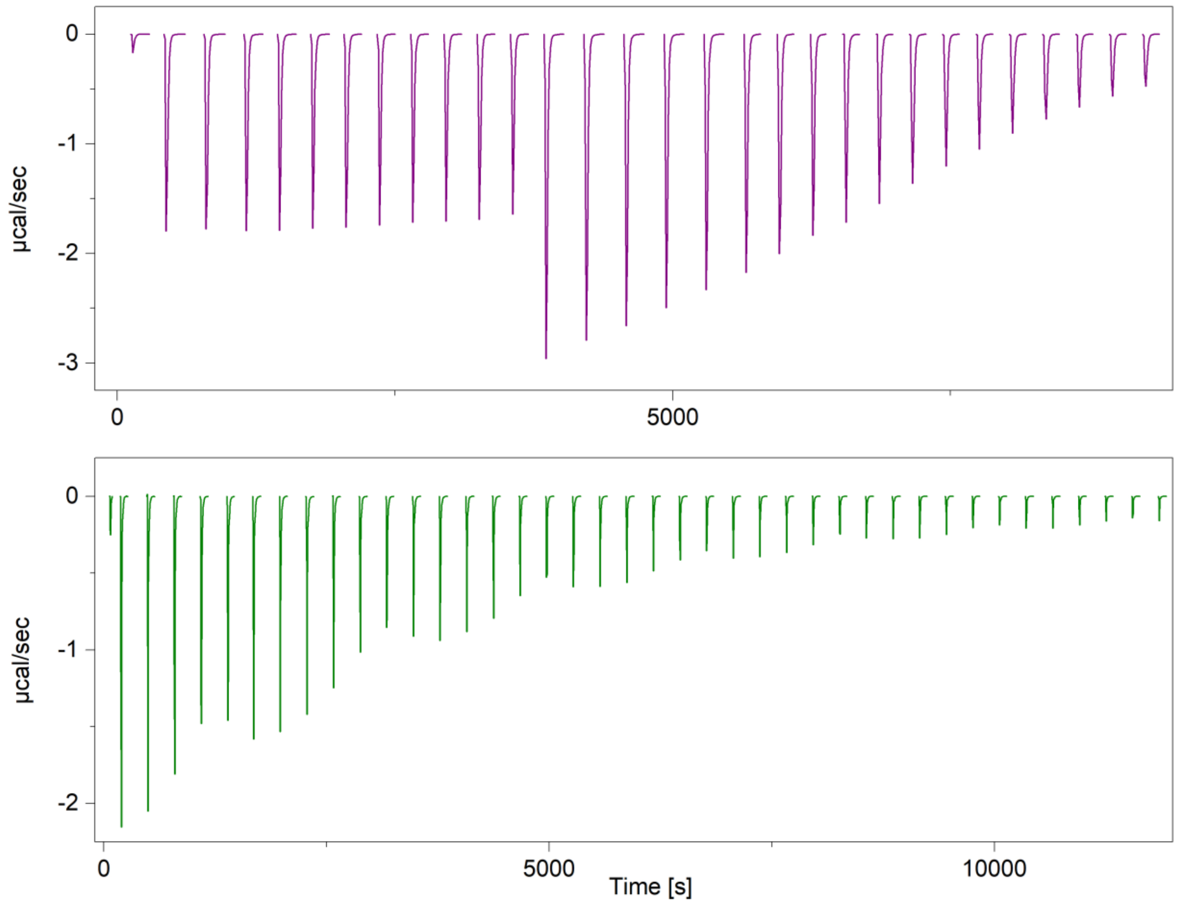

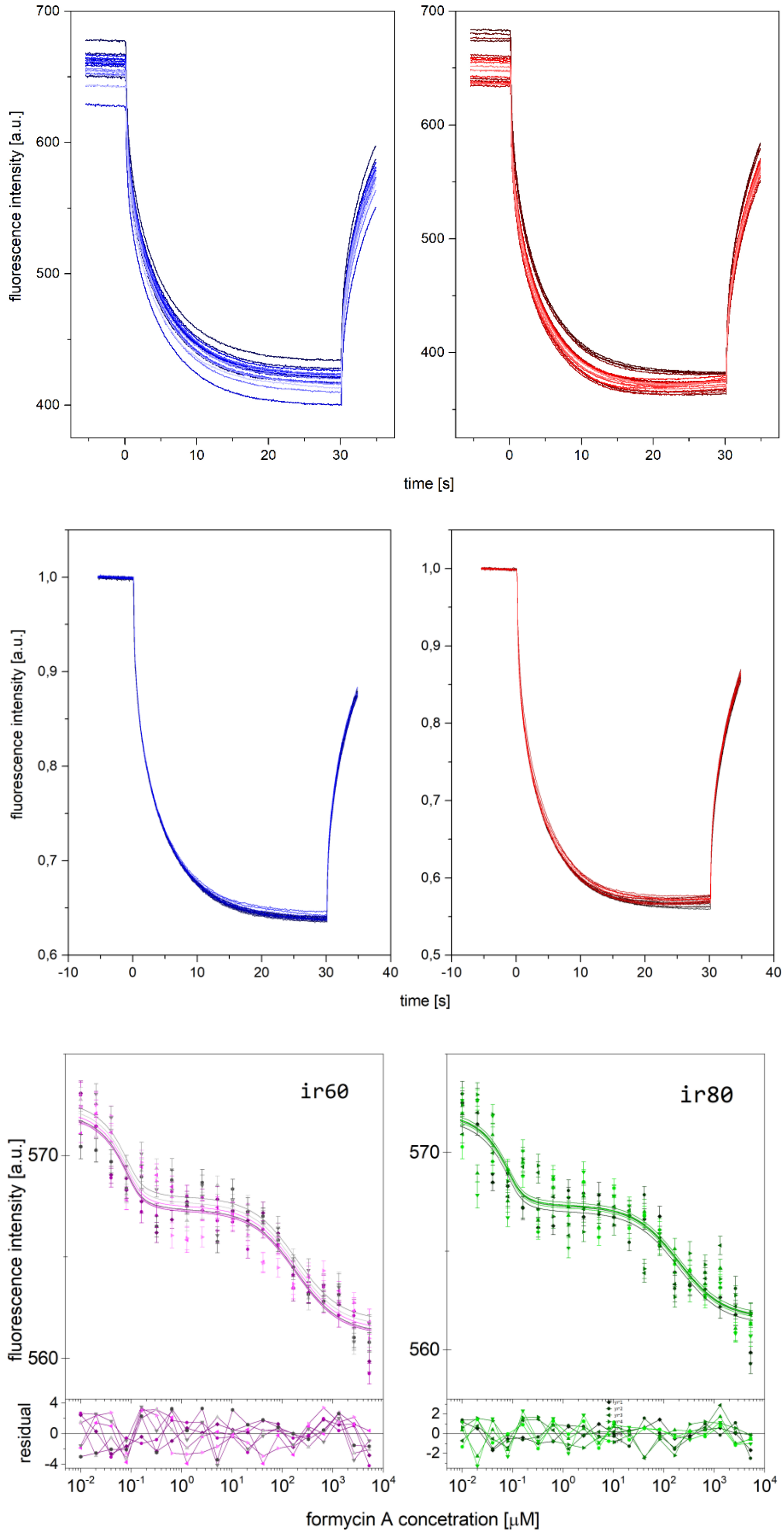

Figure S7

The representative series of raw MST curves (upper panels), and the same data normalised (middle panels), obtained for the PNP-WT-Pi complex titrated with formycin A, at 25°C, in 50 mM Tris/HCl buffer pH 7.6, with the relative heating laser power 60% (left panels) and 80% (right panels). The increase of ligand concentration is marked with the gradient of the colour, black for the highest, blue/red for the lowest. Two lower panels show titration curves obtained from these and other MST experiments for the same complex, conducted in exactly the same conditions, to show that experiments in which 80% relative heating laser power was used give better quality data.

Figure S8

An example of the uniphasic titration curve (WT PNP titration with phosphate, followed by the near-UV CD detection). One-, two-, and three-binding site models fitted are shown as green, violet and red curves, respectively. Respective residual plots are shown on the right, using the same colour as the curve fitted. Although the signal changes monotonically it is clear even on the basis of the visual inspection that the one-binding site model should be rejected. In turn, the residual plots indicate that the two-binding site model should be rejected as well.

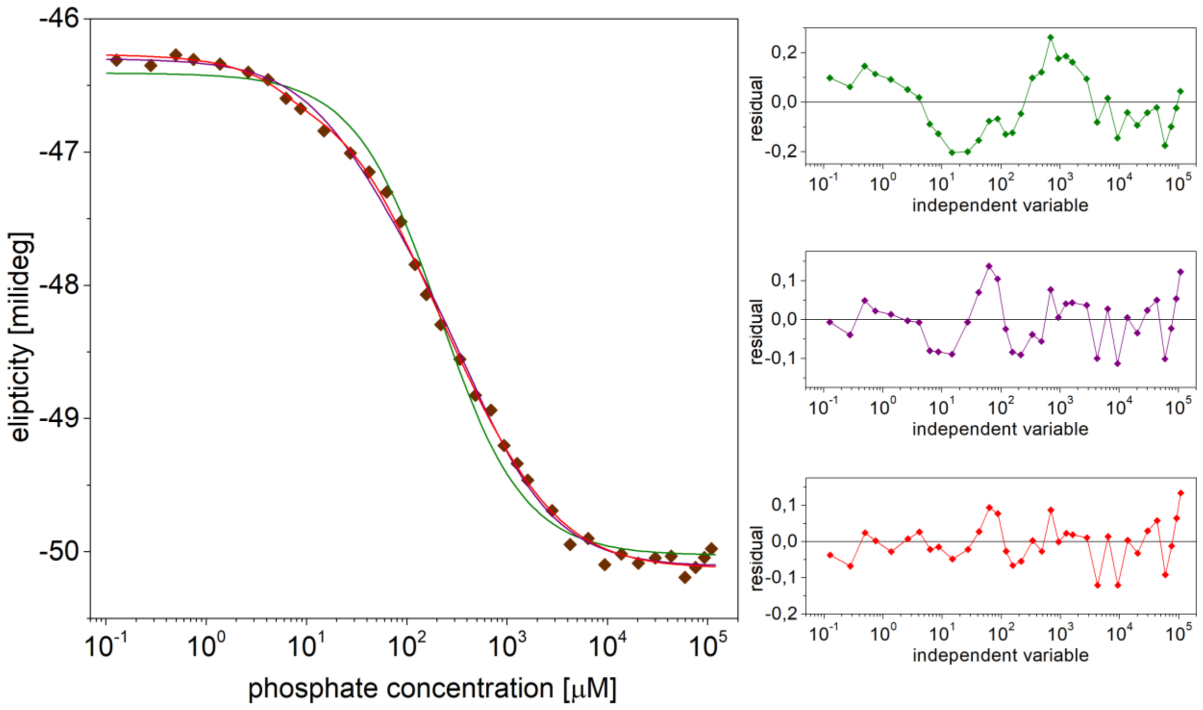

Table S1

Discrimination analysis of the tested models (1bs, 2bs, 3bs denotes one-, two-, and three-binding-site model, respectively) for all titrations described in this study. Probability that observed distribution of runs in residual plots is random from the Wald-Wolfowitz test (WW: p), and weights from the Akaike Information Criterion (AIC: w) are presented. The best model is marked in bold.

Enzyme	titrant	Model	fluorescence titrations	thermophoretic titrations	CD titrations	calorimetric titrations
WT	Pi	1bs<br>2bs<br>3bs	WW: p = 0<br>AIC: w = 0<br>WW: p = $9.12\cdot10^{-5}$<br>AIC: w = 0<br>**WW: p = 0.83**<br>**AIC: w = 1.0**	WW: p = $2.10\cdot10^{-7}$<br>AIC: w = 0<br>**WW: p = 0.729**<br>**AIC: w = 0.776**<br>WW: p = 0.126<br>AIC: w = 0.224	WW: p = 0<br>AIC: w = 0<br>WW: p = $6.95\cdot10^{-7s}$<br>AIC: w = 0<br>**WW: p = 0.255**<br>**AIC: w = 1.0**	
Y160W	Pi	1bs<br>2bs<br>3bs	WW: p = 0<br>AIC: w = 0<br>WW: p = $5\cdot10^{-7}$<br>AIC: w = 0<br>**WW: p = 0.43**<br>**AIC: w = 1.0**	WW: p = 0.100<br>AIC: w = 0.202<br>**WW: p = 0.586**<br>**AIC: w = 0.721**<br>WW: p = $3.64\cdot10^{-4}$<br>AIC: w = 0.077	WW: p = 0<br>AIC: w = 0<br>WW: p = 0.099<br>AIC: w = 0<br>**WW: p = 0.852**<br>**AIC: w = 1.0**	
WT/Pi	FA	1bs<br>2bs<br>3bs		WW: p = $8.89\cdot10^{-7}$<br>AIC: w = 0<br>**WW: p = 0.131**<br>**AIC: w = 0.904**<br>WW: p = 0.002<br>AIC: w = 0.060		WW: p = 0.0029<br>AIC: w = 0<br>WW: p = 0.48<br>AIC: w = 0<br>**WW: p = 0.69**<br>**AIC: w = 1.0**
Y160W/ Pi	FA	1bs<br>2bs<br>3bs		WW: p = 0<br>AIC: w = 0.000<br>WW: p = 0.103<br>AIC: w = 0.008<br>**WW: p = 0.592**<br>**AIC: w = 0.992**		WW: p = 0<br>AIC: w = 0<br>WW: p = 0.00025<br>AIC: w = 0<br>**WW: p = 0.049***<br>**AIC: w = 1.0**

* 0.05 is a boundary value, i.e. for $p < 0.05$ the hypothesis that the observed distribution of runs in residual plots is random should be rejected.
